# The Improvement Default: People Presume Improvement When Lacking Information

**DOI:** 10.1177/01461672231190719

**Published:** 2023-08-07

**Authors:** James G. Hillman, Jillian P. Antoun, David J. Hauser

**Affiliations:** 1 Queen’s University, Kingston, Ontario, Canada

**Keywords:** improvement, change, judgment and decision-making, temporal self appraisals, unrealistic optimism, negativity bias

## Abstract

People erroneously think that things they know little about improve over time. We propose that, due to salient cultural narratives, improvement is a highly accessible expectation that leads people to presume improvement in the absence of diagnostic information. Five studies investigated an improvement default: a general tendency to presume improvement even in self-irrelevant domains. Participants erroneously presumed improvement over esoteric historical time periods associated with decline (Study 1). Participants arranged a stranger’s experiences to produce trends of improvement (Study 2). Participants presumed improvement for a fictional city when given no diagnostic information about it (Study 3). Finally, participants who perceived more past improvement were less supportive of policies that may precipitate further improvement (Study 4). Implications for consequences, such as complacency toward improving inequality, are discussed.

People often presume things will get better, even when they have little evidence to support these presumptions. For instance, despite dire prognostications from climate experts, most Americans and Canadians think that global warming will get better (Leger, 2021). Many people have faith that the stock market, despite its short-term volatility, will trend toward growth (Brown, 2014). People, on average, think that most people get wiser and more rational as they age (Hillman & Hauser, 2021). In the present research, we propose that people have an “improvement default.” When lacking information, people by default presume that things improve.

## Presumptions of Improvement and Decline When Information Is Abundant

Research on assessments of change over time has shown that people wrongly perceive both improvement and decline in various domains. When evaluating themselves and close others, people tend to wrongly presume improvement (Busseri, 2022; Choma et al., 2014; Hillman & Hauser, 2021; Lachman et al., 2008; McFarland & Alvaro, 2000; O’Brien & Kardas, 2016; Ryff, 1991; Shepperd et al., 2015). For instance, in retrospective and prospective judgments about oneself, people expect improvement; they see their past self as worse than it was, and they forecast rosy futures for themselves (Peetz & Wilson, 2008; Wilson & Ross, 2001). This tendency is often attributed to self-enhancement and optimism, which encourage beliefs of improvement over time and life stories of growth (Chopik et al., 2020; Tesser, 2000). In general, when making judgments about the trajectory of the self, people confuse hopes with expectations and wrongly infer/project desired outcomes.

On the contrary, when evaluating non-self social domains, people often wrongly presume decline (Liu & Szpunar, 2023; Pinker, 2018; Shrikanth et al., 2018). In general, people tend to have an unwarranted belief that “things aren’t the way they used to be” (Eibach & Libby, 2009). For instance, Americans tend to believe that several moral domains (e.g., crime) are getting worse over time (Mastroianni & Gilbert, 2023), and people’s assessments of recent trends in social domains are overly pessimistic (Mitchell & Tetlock, 2022).

An important factor guiding these different presumptions is knowledge and information. Prior research on assessments of change have focused upon domains for which people have either relevant information or salient exemplars. When making judgments about the self, people can leverage their self-schemas or memories. When making judgments about other individuals, people apply beliefs about trait-relevant information (e.g., Choma et al., 2014; Hillman & Hauser, 2021). When making judgments about group-level social domains like morality and crime, people may call to mind highly accessible events (e.g., Lichtenstein et al., 1978; Mastrorocco & Minale, 2018).

People do not always have relevant information though, and often render confident judgments despite lacking information. Many will reach confident (inaccurate) conclusions about probabilistic relationships after recruiting inadequate amounts of evidence (Klein & O’Brien, 2018) or prior to viewing any evidence at all (Sanchez & Dunning, 2018). Most people will quickly form lasting impressions of others from minimal samples of personal information (e.g., a 30 second clip of a lecture, Ambady & Rosenthal, 1993). Little research has assessed how people presume change in the absence of salient information. However, this knowledge is important for understanding the early-stage default intuitions about change that exist prior to evidence recruitment or information consideration. These early-stage intuitions are important as they may guide subsequent information recruitment or consideration. We suggest that even in the absence of immediately salient diagnostic information, there is a culturally universal source of information to which deciders may default.

## Narratives of Improvement

Master narratives are cultural beliefs for the trajectory of change over time for various domains (McLean & Syed, 2015). These narratives serve as an indicator for what change is typical and what one could expect to happen in the future. Narratives impact several psychological processes. For instance, restorative narratives (i.e., stories focusing on recovery after difficulty) foster more positive attitudes toward refugees (Paravati et al., 2022). Rags-to-riches narratives (i.e., stories wherein an impoverished protagonist achieves solvency/wealth) increase internal attributions of wealth (Kim, 2023). By providing easily accessible exemplars of change, narratives form expectations that are projected onto novel evaluations of the self and others (Hillman & Hauser, 2021).

A variety of qualitatively different narratives may permeate a culture (McLean & Syed, 2015). However, in North America, many narratives share a common trajectory: improvement. Narratives of improvement have surged in popularity in television (e.g., Kim, 2023). Film demonstrates a similar trend; the North American box office top grossing films are dominated by stories where the protagonist struggles against the odds and triumphs (e.g., *Avengers, Avatar*, and *The Lion King*). Many of these stories utilize a common framework used across time and culture: the hero’s journey (Campbell, 1949). This narrative is characterized by improvement wherein the protagonist struggles before reaching victory. Indeed, given this ubiquity, it is no coincidence that research on narratives tends to focus on narratives of improvement (e.g., rags-to-riches, Kim, 2023; redemption, Perlin & Fivush, 2021; restoration, Paravati et al., 2022).

Improvement is a dominant feature of stories in contemporary North American culture. The ubiquity of narratives that “things improve over time” may affect how people think about change for unfamiliar things that have no relevance to themselves. Chronically accessible concepts often provide automatic responses to stimuli (Higgins, 1996; Kahneman, 2011). As such, we propose that, consistent with the abundance of stories that highlight improvement, people tend to hold a default intuition that all things typically improve over time. When lacking other diagnostic information, people will apply salient cultural narratives of improvement to their judgments.

## The Intersection of the Improvement Default With Other Presumptions of Change

The idea that “things improve over time” dominates Western narratives about the trajectories of change (Campbell, 1949). We propose that improvement is a chronically accessible concept that serves as a default for evaluations of change over temporal sequences. People only apply accessible concepts to ambiguous judgments (Anderson, 1971; Higgins, 1996). As such, we posit that, when projecting how a target will change in the absence of diagnostic evidence, a decider will default to the intuition that the target did (or will) improve, and the improvement default will emerge. Like other intuitive responses (Kruger et al., 2004; Todorov et al., 2002), this intuition should be overridden by other accessible diagnostic information.

When evaluators have diagnostic evidence of change, other mechanisms are likely to encourage presumptions of improvement or decline (see Table 1). When evaluating selves or self-relevant domains, self-enhancement and trait optimism encourage presumptions of improvement. People have ample evidence to draw upon for self-related judgments (memories, trajectories, etc.) and are motivated to see themselves in a positive light (e.g., Tesser, 2000). Thus, in these situations, people may not draw upon an improvement default as they have salient (albeit highly biased) information that suggests improvement. In addition, when evaluating mixed evidence (i.e., evidence of both improvement and decline), negativity dominance (Baumeister et al., 2001; Rozin & Royzman, 2001) and asymmetric tipping points (O’Brien & Klein, 2017) likely lead people to overemphasize declining (negative) evidence and underemphasize improving (positive) evidence. In these situations, the improvement default should not emerge; instead, negativity dominance should encourage presumptions of decline.

**Table 1:** Theoretical Nomological Network Disambiguating Mechanisms of Change.

Judgment domain	Theoretical mechanism	Judgment outcome
Self-related judgments	Self-esteem maintenance (Tesser, 2000)	Improvement
Non-self-related judgment		
No information	Improvement default	Improvement
Mixed information	Negativity dominance (O’Brien & Klein, 2017; Rozin & Royzman, 2001)	Decline

To provide evidence for the improvement default in the following studies, we focus upon judgments about relatively esoteric targets for which people lack diagnostic evidence. We use judgment targets that are outgroups, or entirely fictional, to minimize what self-related information participants may leverage. In several studies, we manipulate evidence by providing or withholding it to track the emergence of the improvement default as a function of information. We also focus upon non-self-relevant judgments to disentangle this tendency from motivational mechanisms such as self-enhancement, as research has found that temporal self-enhancement should not apply to outgroups (e.g., Hillman & Hauser, 2021, for “average other” or Wilson & Ross, 2001, for “acquaintance”). Finally, we also use statistical controls for the potential influence of dispositional optimism.

## The Present Research

In five studies (*N* = 1,286) and six supplemental studies^1^ (*N* = 1,521), we investigated whether people demonstrate a default toward expecting improvement in evaluations when they lack diagnostic evidence. To mitigate the potential influence of self-enhancement and optimism on people’s relevant information, we selected historical and non-self-relevant (i.e., outgroup) judgment targets (e.g., foreign countries, strangers, novel cities).

In Studies 1a and 1b, we assess whether participants default to expect improvement in esoteric historical domains (e.g., Happiness of Romans from 210 to 410) and whether that default is abated by the inclusion of diagnostic information (e.g., Decline and Sacking of Rome). Study 2 evaluates whether participants default to expect improvement for an unknown individual when not explicitly prompted to consider change over time. Study 3 investigates whether defaults of improvement about an unknown fictional city depend upon the absence of diagnostic information. Finally, Study 4 demonstrates implications of an improvement default by assessing whether perceptions of societal improvement (e.g., improvements in equality) make people more complacent about social measures that might precipitate improvement (e.g., diversity initiatives).

For all Mechanical Turk studies, we used Cloud Research Approved participants (Hauser et al., 2022) to ensure data quality and included a comprehension check to ensure participants read and understood the studies. We report how we determined sample size as well as all data exclusions, manipulations, conditions, and measures. Additional studies and analyses can be found in the supplemental documents. Preregistration, data, materials, and analysis code are available at OSF.^2^

## Study 1a

A theme across narratives in North America is improvement. The ubiquity of this theme may foster a default belief that the trajectories of most domains bend toward improvement. As such, when evaluating whether an unknown domain improves/declines over time, people may have a default and highly accessible intuition of improvement.

In Study 1a, participants evaluated how several domains had changed over historical periods (e.g., “Happiness for Romans in Rome from 210 AD to 410 AD”). Domains were ones associated with historical decline; for instance, 210 AD to 410 AD exhibited decline for the Roman Empire as Romans suffered from invasion, disease, and famine, culminating in the 3-day-long sacking of Rome in 410 (Heather, 2006). Half of the items contained additional information diagnostic of decline (e.g., “Happiness for Romans in Rome from 210 AD to 410 AD (the decline and sacking of Rome)”) while the other half did not.

We expected the improvement default to manifest only for ambiguous judgments (i.e., judgments lacking diagnostic information; Kruger et al., 2004; Todorov et al., 2002). Thus, we hypothesized that participants would (erroneously) expect improvement for items containing no additional diagnostic information, and participants would (accurately) not expect improvement for items containing additional diagnostic information.

### Method

#### Participants

A power analysis indicated 180 participants would be sufficient to reach 80% power for a small within-between analysis of variance (ANOVA) interaction (η^2^ = .02), assuming minimal correlation (*r* = .10) between measures. As such, we recruited 211 American participants from Amazon’s Mechanical Turk in exchange for US$45 cents. We removed 19 participants for failing the comprehension check. Of the remaining 192 participants, 123 (64%) identified as male and 69 (36%) identified as female. Participants’ ages ranged from 19 to 72 (*M* = 37.11, *SD* = 10.54).

#### Materials and Procedure

Participants viewed eight timeframes in which historical decline occurred (e.g., Rome from 210 to 410), and evaluated a relevant domain for each timeframe (e.g., happiness for Romans). Half of the items contained diagnostic information in the form of a concise “hint” about what historically happened in that timeframe (e.g., “the decline and sacking of Rome”) while the other half did not.

We split the eight items into two blocks, each with four items (Block A and Block B). We used Block (A vs. B) as a between-subjects variable determining which items contained diagnostic information and which items did not. That is, for participants assigned to items with information in Block A (B), items in Block A (B) contained extra information while items in Block B (A) did not. As such, each participant was exposed to half of the timeframes with information and half with no information (creating a within-subjects factor of information), and which particular items had information was a between-subjects blocking factor. For each item, participants rated whether the given domain had improved or declined during the given time frame on a 1 (*greatly declined*) to 7 (*greatly improved*) scale. To avoid participants feeling obligated to use the entire scale, participants also completed two filler items where historical improvement was likely to have occurred (e.g., health in Europe from 1941 to 2011). Responses to these items were not analyzed.

### Results and Discussion

To assess whether people default to assuming improvement for historical time frames when they lack information, we used a 2 between (block: A vs. B) by 2 within (information: information vs. no information) mixed model ANOVA on ratings of improvement/decline. As hypothesized, the main effect of information was significant, *F*(1, 190) = 62.11, *p* < .001, η^2^ = .25, 95% confidence interval (CI) = [.15, .34]. Domains without diagnostic information were seen as improving (*M* = 4.46, *SD* = 0.98) to a greater degree than domains with diagnostic information (*M* = 3.82, *SD* = 1.15). The main effect of block was also significant, *F*(1, 190) = 6.96, *p* = .009, η^2^ = .04, 95% CI = [.01, .10]. There was also a significant interaction between information and block, *F*(1, 190) = 14.65, *p* < .001, η^2^ = .07, 95% CI = [.02, .15].

**Figure 1.**
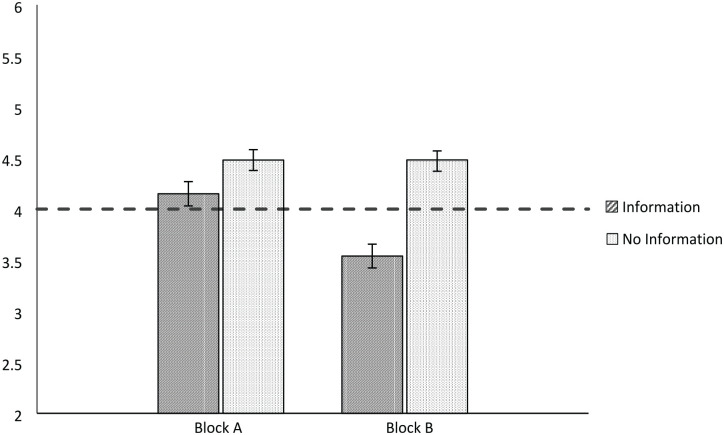
Perceptions of Change by Block and Information. *Note.* Values greater than 4 indicate improvement and values less than 4 indicate decline. Error bars indicate standard error.

Our critical hypotheses concerned whether the no information condition yielded significantly higher ratings of change (i.e., more improvement) than the information condition in each block. For Block A participants, there was a significant difference between items with information (*M* = 4.15, *SD* = 1.08) and items without information (*M* = 4.48, *SD* = 0.87), *t*(92) = 3.27, *p* = .001, *g* = 0.34, 95% CI = [0.13, 0.55]. Similarly, for Block B participants, there was a significant difference between items with information (*M* = 3.51, *SD* = 1.13) and items without information (*M* = 4.45, *SD* = 1.07), *t*(98) = 7.55, *p* < .001, *g* = 0.76, 95% CI = [0.53, 0.98]. Thus, the interaction suggests that the no information condition yields significantly higher ratings of improvement in both blocks, but the effect is stronger for one set of items than the other. See Figure 1.

To assess whether a given domain is actually improving or declining, we used one-sample *t* tests to assess whether perceptions were significantly different from the middle point (4 = “no change”). In line with the improvement default, domains without information were seen as improving (*M* = 4.46, *SD* = 0.98), *t*(192) = 6.58, *p* < .001, *g* = 0.48, 95% CI = [0.33, 0.62]. When given information, participants rated domains as declining (*M* = 3.82, *SD* = 1.15), *t*(192) = −2.16, *p* = .031, *g* = −0.16, 95% CI = [−0.30, −0.01]. See Figure 1.

Generally, people seem to default to assuming improvement for unfamiliar historical time frames. Like many intuitions, the improvement default is endorsed when judgments are lacking diagnostic information. However, this default is abandoned when information relevant to decline is salient. That is, people generally expect improvement and attenuate this expectation when given information that suggests otherwise.

However, this study is not without limitations. First, in this study (and in many discussed in the supplement), participants may simply be biased to use higher numerical scale responses when lacking diagnostic information, inflating their responses to items with no information. In addition, the combined presentation of both items with and without information may have encouraged participants to contrast them, resulting in more perceived improvement for the items without information. Finally, participants were not incentivized for accuracy, so they may have endorsed optimistic responses of what they wished had happened when lacking firm evidence to the contrary. Study 1b aims to address these limitations.

## Study 1b

While Study 1a provided some evidence of our hypothesized effect, it had several limitations. For Study 1b, we aimed to address each of these limitations and replicate our Study 1a results with a preregistered study.^3^

### Method

#### Participants

Power analyses based on the results of Study 1a indicated that as few as 100 participants per condition (200 total) could maintain acceptable levels of power (80%). However, we opted to recruit additional participants to account for any removed due to exclusion criteria (i.e., failing comprehension checks). As such, we recruited 300 American participants from Amazon’s Mechanical Turk in exchange for US$45 cents. We removed 25 participants for failing to complete the comprehension check. Of the remaining 275 participants, 156 (57%) identified as men and 115 (42%) identified as women, three participants identified as non-binary (1%), and one declined to answer. Participants’ ages ranged from 21 to 77 (*M* = 39.97, *SD* = 11.72) years.

#### Procedure and Materials

As with Study 1a, the survey asked participants to indicate whether domains changed over historical time periods. The procedure and items were identical to Study 1a, except for a few key changes. First, the endpoints of the rating scale were inverted to account for any potential bias to endorse higher numbers when lacking diagnostic information. Thus, ratings of change were made on 1 (*greatly improved*) to 7 (*greatly declined*) point scales with a neutral option at 4 (*no change*).

Second, we manipulated information condition (information vs. no information) to be randomly assigned between subjects to account for any potential contrast effects that could arise from a within-subjects presentation. Thus, half of the participants received all items with diagnostic information indicative of decline while the other half received all items with no additional diagnostic information. Because of the lack of a within-subjects variable, the Blocking variable was also removed.

Third, we incentivized participants to be accurate in their ratings. We instructed participants that they had an “opportunity to WIN A BONUS OF $50!!” Participants were told that 10 historians rated these same time periods, and the participant whose ratings were closest to the experts’ consensus would receive a $50 USD bonus.

Finally, we included a measure of dispositional optimism (Scheier et al., 1994). This measure is composed of three positively coded items (e.g., “In uncertain times, I usually expect the best”), three negatively coded items (e.g., “If something can go wrong for me, it will”), and four filler items. Participants rated how much they felt the items applied to them on a 1 (*strongly disagree*) to 7 (*strongly agree*) scale. Non-filler items had good reliability (α = .92), so we averaged them for a composite dispositional optimism index.

### Results and Discussion

For clarity and consistency with other studies, we reverse coded change ratings in this study, so high numbers indicated improvement and low numbers indicated decline (although, note that, in the actual procedure, lower numbers indicated improvement and higher numbers indicated decline).

Do people default to assuming improvement for historical time frames when they lack information? As predicted in our preregistration and consistent with Study 1a, participants who had no diagnostic information (i.e., no information; *M* = 4.53, *SD* = 1.01) reported more improvement across domains than those who received diagnostic information (i.e., information; *M* = 3.71, *SD* = 1.09), *t*(273) = 6.47, *p* < .001, *g* = 0.78, 95% CI = [0.53, 1.02].

**Figure 2.**
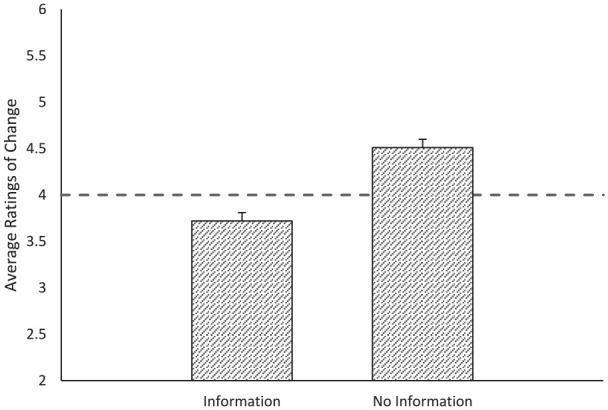
Average Change Ratings by Information Condition. *Note.* Error bars indicate standard error.

As with Study 1a, we used one-sample *t* tests to assess whether perceptions were significantly different from the middle point (4 = “no change”). In line with the improvement default, and consistent with our second preregistered hypothesis, participants who assessed domains with no information rated them as improving (i.e., mean change higher than midpoint) across domains *t*(135) = 5.93, *p* < .001, *g* = 0.51, 95% CI = [0.33, 0.69]. In addition, participants who assessed domains with information rated them as declining, *t*(138) = 3.26, *p* = .001, *g* = 0.28, 95% CI = [0.11, 0.45]. See Figure 2. Like Study 1a, people defaulted to expect improvement across esoteric historical time points where decline was present unless they were given information that suggested otherwise.

We then probed whether dispositional optimism accounted for the observed improvement default. There was a small positive correlation between dispositional optimism and ratings of change over all time periods, *r*(275) = .18, *p* = .002. Increased optimism corresponded with ratings of more improvement. To be sure optimism was not driving our observed effects, we repeated the previous analyses using an analysis of covariance (ANCOVA), with dispositional optimism entered as a covariate. In line with our preregistered hypotheses, the effect of information remained significant when controlling for dispositional optimism, *F*(1, 272) = 38.34, *p* < .001, η^2^ = .12, 95% CI = [.06, .20]. Thus, while optimists do tend to forecast more improvement, dispositional optimism is not a sufficient explanation for why people demonstrate an improvement default when lacking information.

Overall, this preregistered study corroborates the results of Study 1a while addressing its limitations. Generally, across domains, people tend to feel things improve over time when they lack information. However, information suggesting decline can sharply attenuate these beliefs. Study 1b rules out several alternative potential explanations for the observed improvement default, including method variance (i.e., scale direction, within vs. between design), lack of motivation (i.e., no incentives to respond accurately), and individual differences (i.e., optimism). However, we still directly ask participants about change, which could be leading them to think of improvement and limit the generalizability of the effect. We aim to address this in Study 2.

## Study 2

Study 2 examined whether people infer improvement for unknown targets even when no prompts to evaluate change are given. To assess this, we asked participants to order the events of an unknown person in a chronological order and then rate the positivity of each event. Participants were asked to order events across either 5 days or 5 weeks. We expected that events would be ordered such that they demonstrated a linear trend of positivity increasing over time (i.e., a significant positive within-subjects linear effect of time), regardless of the timeframe.

### Method

#### Participants

Power analysis indicated that to reach 90% power to detect small within-subjects differences in each condition (η^2^ = .01), we would need 215 participants (assuming average correlations between measures; *r* = .30) in each condition. Because we tested two types of time frames, we doubled this estimate and recruited 436 from Mechanical Turk in exchange for US$45 cents. We removed 30 participants for failing the comprehension check or providing incomplete data. Of the remaining 406 participants, 225 (55%) identified as male, 171 (42%) identified as female, and 10 participants identified as non-binary or did not disclose their preferred gender identity. Participant ages ranged from 19 to 75 (*M* = 39.17, *SD* = 11.64) years.

#### Procedure and Materials

Participants were randomly assigned to a time frame of a month or a week (between-subjects) and then were introduced to a fictional individual named “Denise.” Instructions explained that on each of the 5 days (weeks) of Denise’s work week (month), one of five events happened to her in the parking lot. Participants were shown two positive events (e.g., “Denise got to pet a cute dog”), two negative events (e.g., “A driver in the parking lot yelled at Denise”), and one neutral event (e.g., “Denise left a note in her car to pick up the mail after work”). We asked participants to order the events in the way they felt they occurred over the course of the week (month). After ordering events, participants then rated the events in positivity on a 1 to 7 scale, where 1 indicated *very negative* and 7 indicated v*ery positive.* We calculated the positivity of each day (week) as the positivity rating that each participant assigned to the event which they designated as occurring on that timepoint. This yielded an ordered set of 5 days (weeks), each with its own positivity score, for each participant.

### Results and Discussion

We used a mixed-model ANOVA to assess whether there was a difference among positivity ratings across the five time points (time point: 1, 2, 3, 4, 5) and whether this varied based on the time frame used (days vs. weeks). There was a main effect of time point on the ratings of positivity, *F*(4, 1612) = 13.16, *p <* .001, η^2^ = .03, 95% CI = [.02, .05]. There was no significant main effect of time frame, *F*(1, 403) = 2.43, *p =* .120, η^2^ = .01, 95% CI = [.00, .03]. There was a small interaction between time points and time frame, *F*(4, 1612) = 2.59, *p =* .035, η^2^ = .01, 95% CI = [.00, .01]. To decompose this interaction, we assessed the between-subjects difference at each time point using Bonferroni corrections to control for multiple comparisons. The only significant difference was the fourth time point, which was significantly less positive in ratings of weeks (*M* = 3.69, *SE* = 0.19) than days (*M* = 4.34, *SE* = 0.16), *p* = .009. All other time points were not significantly different from one another (all *p*s > .100).

**Figure 3:**
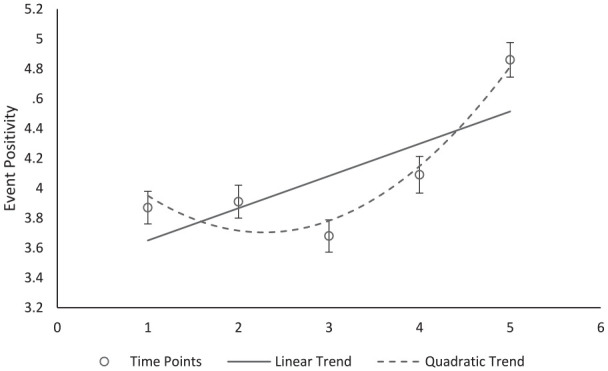
Mean Positivity Rating for Selected Events by Time Point. *Note.* Error bars indicate standard error.

Our main hypothesis was that participants would presume improvement in events over the progression of the ordered time points. To assess this, we conducted linear and quadratic contrasts for the main effect of time point. Both the linear contrast, *F*(1, 403) = 19.21, *p* < .001, η^2^ = .046, 95% CI = [.01, .09], and quadratic contrast, *F*(1, 403) = 25.52, *p* < .001, η^2^ = .060, 95% CI = [.02, .11] were significant. Neither contrast significantly interacted with time frame (*p*s > .100), suggesting that participants applied the same pattern to their ratings regardless of time frame. As shown in Figure 3, there were both significant linear and quadratic trends across both conditions (see the supplemental analyses for individual means and comparisons).

As suggested by the improvement default, results demonstrated a significant linear trend; participants presumed that events improved from the beginning to the end of the time period. These results were consistent regardless of whether the time frame presented was 5 days or 5 weeks. This suggests that people have an intuition of how change occurs and then map that onto different time periods when lacking other information.

There was also an unexpected quadratic effect suggesting more curvilinear presumptions of improvement, which start relatively flat then increase substantially in the end. While we did not hypothesize this curvilinear relationship, this does reflect the narrative trajectories of the hero’s journey (e.g., things often get worse before they get better; Campbell, 1949). While the beginning to end of the sequence demonstrates improvement (consistent with Studies 1a and 1b), the shape of that improvement may be more complex than a simple linear trend when people are able to make more granular forecasts.

Overall, the results of Study 2 support the hypothesized improvement default. Furthermore, this study provides evidence that this default emerges even when participants are unprompted to think about change over time. Taken together with the results of Study 1a and 1b, this suggests a relatively robust tendency for individuals to anticipate improvement when they have no relevant evidence of such and no self-interest to do so.

## Study 3

The prior studies suggest that people hold an improvement default and presume that unknown domains improve. While people could (and perhaps should) assume no change (Ross, 1989), they instead default to improvement, even when this assumption is incorrect and accuracy is incentivized. Default intuitions are typically endorsed when diagnostic information is unavailable (Kruger et al., 2004; Todorov et al., 2002). However, default responses are abandoned in the presence of any diagnostic information. If responses that things improve stem from endorsing an improvement default, this suggests that people should not respond that things improve when *any* diagnostic information about decline is available. Thus, the presence of mixed diagnostic information about a judgment target (i.e., both improvement and decline information) should eliminate the tendency for people to endorse an improvement default.

Other mechanisms should influence non-self-relevant judgments when mixed information is provided. Negative information tends to outweigh positive information when both are present and equal in magnitude (O’Brien & Klein, 2017; Rozin & Royzman, 2001). Thus, when mixed information is provided, the improvement default should not emerge. Negative information should dominate, and people should presume decline.

In Study 3, we sought to provide empirical evidence disambiguating the improvement default from mechanisms that influence evaluations of change over time. Participants evaluated whether a city was improving, remaining stable, or declining. General information about the city was provided and additional information diagnostic of improvement and/or decline was manipulated. This allowed complete control over participants’ a priori knowledge of the topic (a limitation of Study 1a and 1b). Participants received additional information about (a) the city’s improvement, (b) the city’s decline, (c) the city’s improvement and its decline (i.e., mixed information), or (d) no additional information at all. Improvement/decline information was pilot tested to have similar strength of impact on evaluations when presented in isolation.

If the improvement default depends upon a lack of diagnostic evidence, then participants should be hesitant to endorse this default when *any* information diagnostic of decline is provided. In this situation, participants should only rate the city as improving when (a) improvement information is provided and when (d) no information is provided. If the presence of diagnostic evidence instigates alternate processes, then negativity dominance should occur when mixed diagnostic information is provided. If this is the case, participants should rate the city as declining when (b) decline information is provided and (c) when mixed information is provided.

### Method

#### Participants

In previous studies, we found large effect sizes (η^2^ = ~.20). However, most of those involved within-subjects designs, so we assumed only a moderate effect size using a between-subjects design. As such, we computed the desired sample size needed to reach 95% power with an η^2^ of 0.06. While this suggested a total sample of 276 would be sufficient, we also wanted to maximize power for pairwise comparisons, so we recruited 400 American participants from Mechanical Turk in exchange for US$45 cents. We removed seven participants for failing to complete the comprehension check. Of the remaining 393 participants, 211 (54%) identified as male, 175 (44%) identified as female, and seven participants identified as non-binary or did not disclose their preferred gender identity (2%). Participant ages ranged from 18 to 79 (*M* = 39.42, *SD* = 11.93) years.

#### Procedure and Materials

Participants read general information about a fictional city named Avalon before receiving additional information about the city in one of four information conditions (randomly assigned between subjects): only improvement information, only decline information, both improvement and decline information (i.e., mixed information), or no change information. Participants assigned to the improvement condition read information such as “The current mayor receiving little to no competition perhaps stems from the fact that people are increasingly happy with his policies in recent years.” Participants assigned to the decline condition read information such as “As local manufacturing moves overseas, Avalon seems to be entering a minor economic recession, with exporting becoming less crucial.” Participants assigned to the mixed condition read a combination of both passages, while participants assigned to the no change information received no additional information.

Improvement and decline information were pretested and selected so that the impact of the information on presumptions of improvement/decline, when presented in isolation, was the same strength (i.e., the absolute value of mean improvement/decline for each were not significantly different). Thus, participants receiving the improvement information should have perceived Avalon to be improving to the same degree that participants receiving the decline information perceived Avalon to be declining. Pretesting analyses and data can be found on our OSF.

For participants receiving both improvement and decline information, the order of presentation was counterbalanced between subjects to eliminate any potential effects of order.^4^ After participants read all information about Avalon, we asked them to rate whether they felt “Avalon is improving or declining in recent years,” on a 1 to 7 scale, where 1 indicated (*greatly declining*), 4 indicated (*no change*), and 7 indicated (*greatly improving.*)

### Results and Discussion

**Figure 4.**
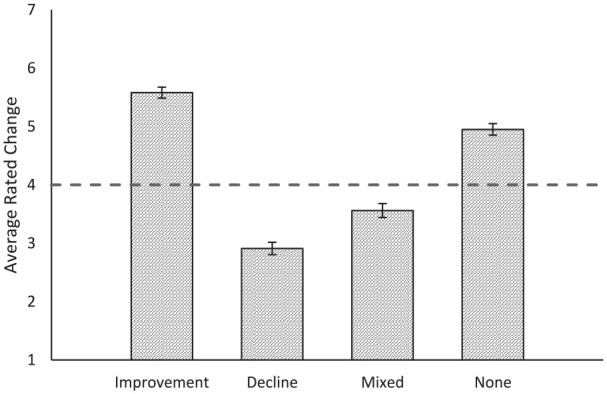
Perceptions of Avalon’s Recent Change by Condition. *Note.* Values greater than 4 indicate improvement and values less than 4 indicate decline. Error bars indicate standard error.

We conducted a one-way ANOVA comparing ratings of perceived change across the four levels of the between-subjects information conditions (growth, decline, mixed, no info). The omnibus effect was significant, *F*(3, 390) = 144.32, *p* < .001, η^2^ = .53. To follow up, we assessed pairwise comparisons between each condition using a Bonferroni correction. All pairwise comparisons were significant (all *p*s < .001). See Figure 4 for mean values.

We compared each information condition against the scale midpoint (4) using one-sample *t* tests to determine whether change was perceived to be improving, declining, or stable. In support of the improvement default, when lacking information (*M* = 4.93, *SD* = 0.99), participants presumed that Avalon was improving, *t*(98) = 9.32, *p* < .001. Confirming that the improvement default is abandoned in favor of negativity dominance when mixed information is provided, mixed information (*M* = 3.57, *SD* = 1.18) led to presumptions that Avalon was declining, *t*(95) = −3.47, *p* = .001. As a manipulation check, the improving information condition (*M* = 5.57, *SD* = 0.94) presumed that Avalon was improving, *t*(99) = 16.72, *p* < .001, and the declining information (*M* = 2.84, *SD* = 1.01) condition presumed that Avalon was declining, *t*(96) = −11.39, *p* < .001.

Study 3 disambiguates the improvement default from other processes relating to judgments of change. The results suggest that the improvement default occurs when evaluators lack diagnostic information about the non-self-relevant judgment targets. Participants inferred sizable improvement when no information was provided. However, when other information was available, the improvement default was abandoned in favor of other mechanisms that guide information use. When mixed improvement and decline information were provided, responses of improvement did not emerge. This is notable because this information was individually pretested to be equivalent in strength. When presented in isolation, improvement information caused participants to rate Avalon as improving to a similar extent that decline information caused participants to rate Avalon as declining. However, when both improvement and decline information were mixed and presented simultaneously, participants rated Avalon as declining, conceptually replicating the effects of asymmetric tipping points on evaluations of change (O’Brien & Klein, 2017).

These results also provide an account of how people can generally seem to be both erroneously optimistic (e.g., myths of racial progress; Richeson, 2020) and erroneously pessimistic (e.g., myths of moral decline; Pinker, 2018). When no information is available, people default to narratives and presume improvement (instead of stability); however, when mixed information is available, negativity dominates, and people presume decline (instead of stability). We examined the implications of these erroneous perceptions of recent change for behavior in Study 4.

## Study 4

Can defaulting to improvement for recent change make one complacent toward actions which may precipitate further improvement? Lacking knowledge of change could lead people to think that things have been improving all along, which may then forestall attempts to further improvement. This would be inconsistent with work that suggests that people often work harder when a goal feels closer (Peetz et al., 2009). However, for goals without a clear endpoint, perceptions of progress may not increase motivation. For example, research has shown that when people perceive large gains in a domain have occurred (e.g., gains in racial equity in wake of the election of the first Black president), they have less support for policies aimed at further gains in that domain (e.g., policies to reduce racial inequality; Esposito & Finley, 2009). This suggests that perceptions of progress do not necessarily increase the motivation to achieve a goal, but rather, in some cases, real improvement may lead people to feel further improvement is not necessary. After all, why would one bother to work on improving something when it has been improving all along?

We tested whether the same was true for inaccurate presumptions of improvement (e.g., uninformed individuals who presume improvement by default). If so, presumptions of improvement in equality stemming from a lack of knowledge about continued racial inequities could make people complacent toward policies that precipitate future improvement in equality. Participants received either no information about changes in equality in the past 20 years or information that equality has stagnated/declined. Participants then rated how equality had changed in the past 20 years, rated how much it would change in the next 20 years, and indicated their support for policies and behaviors that could improve equality in the future. In line with the improvement default, we hypothesized that participants receiving no information would, on average, presume that equality had improved more in the past 20 years compared to those receiving stagnation/decline information. In addition, if the improvement default could stall support for future improvement, then ratings of past improvement in equality should be negatively related to support for future policies aimed at increasing equality. Finally, to rule out optimism as an alternative mechanism underlying these effects, we predicted these patterns would persist even when controlling for dispositional optimism.

### Method

#### Participants

Power analysis indicated that a single predictor model with an *R*^2^ of .05 with 90% power would require ~200 participants. As such, we recruited 244 American participants from Mechanical Turk in exchange for US$45 cents. We removed 21 participants for failing the comprehension check. Of the 223 remaining participants, 119 (54%) identified as male, 99 (44%) identified as female, and four participants identified as non-binary or did not disclose their preferred gender identity (2%). Participant ages ranged from 20 to 76 (*M* = 42.46, *SD* = 12.65) years.

#### Procedure and Materials

Participants were recruited for a survey on perceptions of equality in America. Participants were randomly assigned to receive either no information about equality or receive information diagnostic of stagnation/decline in equality over the past 20 years (e.g., “Black household median wealth is still 10 times less than white households, this gap hasn’t changed in the past 2 decades”). Then, participants rated how much they felt equality has changed in the past 20 years in America on a 1 (*greatly decline*) to 7 (*greatly improved*) scale and how much they thought equality will change in the next 20 years on the same 7-point scale.

Participants then indicated their agreement with supporting five equality-increasing policies on a 1 (*strongly disagree*) to 7 (*strongly agree*) scale (e.g., “Making education more accessible to underrepresented groups, by implementing more scholarship programs specifically for racial minorities”). Next, participants indicated how important seven behaviors are to improve equality on a 1 (*very unimportant*) to 7 (*very important*) scale (e.g., “protesting (lawfully)” or “donating money”). Last, we measured participants’ dispositional optimism, using Scheier and colleagues’ (1994) measure as per Study 1b (α = .90).

### Results and Discussion

#### Improvement Default and Equality

We first assessed whether people felt equality had improved in the past 20 years when they received no additional information about changes in equality. Consistent with prior studies, participants who received information about stagnation/decline (*M* = 4.36, *SD* = 1.68) significantly attenuated their ratings of improvement in equality compared with those who did not receive any information (*M* = 4.82, *SD* = 1.69), *t*(221) = 2.02, *p* = .045, *g* = 0.27, 95% CI = [0.01, 0.54]. In line with the improvement default, when lacking information, people rated equality as significantly improving over the past 20 years (i.e., the mean was greater than the midpoint), *t*(114) = 5.19, *p* < .001, *g* = 0.48, 95% CI = [0.29, 0.68]. Participants still felt there was some improvement in equality even when stagnation/decline information was provided, *t*(107) = 2.23, *p* = .014, *g* = 0.22, 95% CI = [0.02, 0.41].^5^ However, consistent with the improvement default, the absence of diagnostic information amplified perceptions of improvement.

To account for a potential effect of optimism, we submitted ratings of change in past equality to a linear regression, entering optimism and dummy-coded information condition as predictors. Consistent with Study 1b, both information condition, *B* = −0.46, *t*(222) = 2.05, *p* = .041, and optimism, *B* = 0.17, *t*(222) = 2.34, *p* = .020, uniquely predicted people’s perceptions of recent change. Thus, the improvement default remains after controlling for optimism. While optimism does encourage perceptions of improvement, it is not the sole reason why people presume improvement when lacking information.

We next explored whether people felt equality would improve in the next 20 years when they received no additional information about changes in equality (while controlling for optimism). We submitted projections of change in future equality to a linear regression, entering optimism and dummy-coded information condition as predictors. Unlike ratings of past change, information condition did not affect projections of future change, *B* = 0.03, *t*(220) = 0.17, *p* = .869, while optimism did, *B* = 0.23, *t*(220) = 3.43, *p* < .001. Thus, while people presume that equality has recently improved when lacking information, a lack of information does not inflate estimates of future improvement in equality.

#### Presumptions of Improvement and Support for Equity-Improving Policies

We combined ratings of support for future policies (α = .89) into a single index.^6^ Lacking information about equality did not impact support for future policies, *t*(214) = 1.56, *p* = .120, *g* = 0.21, 95% CI = [−0.05, 0.47] for the effect of information condition. However, the information in this condition discusses recent (past) events, and so it may be held as diagnostic in past judgments but not future judgments.

As an alternative test, we assessed whether perceptions of change in equality predicted support for equity-improving policies. Notably, the correlation between perceptions of past change in equality and projections of future change in equality was unexpectedly high (*r* = .59, *p* < .001), suggesting that entering them as simultaneous predictors in a regression would present issues with multicollinearity. Thus, we submitted support for equity-improving policies to two separate linear regressions, the first entering perceptions of past change in equality and optimism as predictors and the second entering projections of future change in equality and optimism as predictors.

Are presumptions of recent improvement in equality associated with declined support for equity-increasing policies? In the first regression, perceptions of recent improvement were associated with reduced support for policies, *B* = −0.24, *t*(220) = −3.68, *p* < .001, while optimism was not associated, *B* = 0.08, *t*(220) = 1.19, *p* = .234. In the second regression, neither forecasts of future improvement, *B* = 0.10, *t*(220) = 1.37, *p* = .173, nor optimism, *B* = 0.02, *t*(220) = 0.29, *p* = .769, predicted support for policies. Thus, even when accounting for optimism, thinking that there have been gains in equality predicted diminished support for future equity-increasing policies. However, forecasting gains in equality does not predict support for future equity-increasing policies. While optimism had small correlations with past (*r* = .15, *p* = .022) and future (*r* = .23, *p* < .001) judgments, like Study 1b, including optimism in each model did not meaningfully change the results.

In sum, these results suggest that people default to assuming recent improvement in equality; that is, when lacking information, they presume that society has gotten substantially more equitable in the last two decades despite this not necessarily being the case (Kraus et al., 2019). In addition, the more that people presumed recent improvement in equality, the less supportive they were toward policies that could improve equality in the future. While optimism was associated with rosier perceptions of recent equality gains, optimism did not impact policy support and could not account for the effect of perceiving improvement on complacency toward policies.

Surprisingly, projections of future change in equality were not affected by a lack of information and did not predict support for equity-increasing policies. There are several factors that may have affected this, such as people’s belief their country should improve (i.e., self-enhancement) or their desire to live in a country which is better in the future. However, it is also important to note that people’s perceptions and forecasts of “improvement” could differ according to personal beliefs; for instance, anti-equality extremists could view a decline in equality as an “improvement.” Furthermore, while there was no effect of information in future projections, this could have been due to provided decline/stagnation information being diagnostic of the past.

## General Discussion

People generally live their lives expecting improvement in various domains (Busseri, 2022; Hillman & Hauser, 2021; Shepperd et al., 2015). While there are many mechanisms that may lead to these expectations, the present research demonstrates that when people lack diagnostic information, they will default to cultural narratives and intuitively assume improvement has occurred. Five studies showed that people, by default, presumed improvement in domains separate from their self and their future when lacking information. In Studies 1a and 1b, participants rated retrospective periods of decline (e.g., the happiness of Romans during the fall of Rome) as having improved when diagnostic information was absent. In Study 2, participants spontaneously presumed improvement over time when asked to order events experienced by another person. In Study 3, participants evaluating the trajectory of a city presumed improvement when lacking diagnostic information (reflective of an improvement default) but presumed decline when information diagnostic of both improvement and decline was present, demonstrating that people abandon the improvement default when information is mixed. Finally, Study 4 demonstrated the implications of the improvement default for public policy; people exhibited an improvement default for recent changes in equality, and people’s tendency to perceive recent improvement in equality predicted less support for policies designed to improve equality.

These studies suggest that people (erroneously) default to feeling improvement has occurred across various domains with little relevance to themselves. Furthermore, this tendency is associated with reduced support for actions that could precipitate further improvement. In a sense, those who feel a great deal of improvement has already occurred (even if they have no evidence to suggest this) seem to feel less of a need for further improvement. However, it is important to note that, while we expect this tendency to apply to both past and future domains, the present research focusses largely on the assessments of the past to limit the potential confounding effects of self-enhancement and optimism. As such, many of our inferences are limited to retrospective judgments. While Study 4 assessed future perceptions of equality, the lack of findings in this domain could be due to the specificity of our provided information (i.e., information regarding past decline isn’t applied to future judgments) or motivational factors relevant to the domain assessed (i.e., people want to feel their country is improving), and so future research should aim to disentangle these many factors potentially influencing future judgments. Study 4 does, however, demonstrate that even if this tendency is constrained to past judgments, these past judgments are still important as they are associated with support for policy in both present and future. Regardless, while the present research provides an account of people’s tendency to default to assume improvement retrospectively, future research could benefit from explorations of whether and how this tendency may function in prospective contexts.

Generally, people often erroneously assume both improvement (e.g., Richeson, 2020; Weinstein, 1980; Wilson & Ross, 2001) and decline (e.g., Eibach & Libby, 2009; Mastroianni & Gilbert, 2023; Pinker, 2018). The current studies suggest that people demonstrate an improvement default and erroneously assume improvement (rather than stability) when lacking diagnostic information. This tendency, however, is dispelled when presented with relevant evidence, and when evidence is mixed, people tend to erroneously assume decline (rather than stability). Of course, because of these necessary conditions, the improvement default is limited to ambiguous domains for which the evaluator lacks immediately relevant diagnostic information. Indeed, an environmentalist who reads about, and researches, environmental decline would be likely to report the environment is in decline because they will have highly salient and accessible diagnostic information. The improvement default is overruled in these circumstances. As such, people are only likely to endorse the improvement default when evaluating a domain about which they are uninformed. As pointed out by Ross (1989), when there is no evidence of change, one *should* assume stability. However, Studies 1a, 1b, and 3 demonstrated that the improvement default reliably leads people to presume improvement when they should not. In addition, many consequential domains in real life are ones where a default assumption of improvement may be incorrect and have negative consequences (e.g., presumptions of gains in racial equality).

### Myths of Progress and Racial Equality

Study 4 demonstrated that perceiving retrospective improvement is associated with complacency toward policies that could precipitate improvement. For instance, the more improvement in equality that people thought had occurred over the past 20 years, the less supportive they were of policies to further improve equality. Thus, the improvement default could contribute to the generation and propagation of myths of progress (e.g., widespread false beliefs of improvement; Kraus et al., 2019; Richeson, 2020). When progress in a domain is stagnant, evidence of change (in any direction) is likely to be scarce, leading people to rely on their default intuitions. Ironically, when lacking evidence of change, people *should* infer stability. However, the results presented here strongly suggest that when lacking evidence of change, people will default to presuming improvement instead.

Issues of social justice present a domain where the improvement default may have far-reaching societal implications. If progress has stalled (or even backslid) in furthering rights for a minoritized group, people in the minority group who experience inequality may have ample evidence of stability or decline, and thus they accurately perceive stability or decline. However, those in the majority group will likely have less direct experiences and thus have less diagnostic information, causing them to default toward presuming improvement. This default presumption of improvement for the majority group may increase their complacency toward these issues and erode support for initiatives because these individuals feel that improvement *has* been happening (despite being uninformed) and feel that no further action is necessary.

Research by Kraus and colleagues (2017) corroborates this supposition, demonstrating that misperceptions about income inequality between White and Black Americans were largest among *wealthy* White Americans (presumably the group with the least direct experience with income inequality). This tendency to assume improvement when it has not been occurring has been applied to Americans’ thinking about racial progress (Richeson, 2020). Contemporary research has assessed some potential factors (e.g., belief in a just world or political alignment, see Kraus et al., 2019; Onyeador et al., 2021). However, the current research suggests that controlling for all other factors, many people may *still* believe progress has happened simply because they lack exposure to evidence that it *has not* happened.

### Conclusion

The present research presented five studies that demonstrated an improvement default: People by default expect improvement over time for domains they know little about, and which have little relevance to themselves. The default reliably appears in evaluations of ambiguous judgment targets. Importantly, when making retrospective judgments, this tendency to presume improvement was associated with complacency toward policies and behaviors which could precipitate actual improvement. In sum, while it may seem wise to assume stability until one has evidence of improvement or decline, people tend to assume improvement until proven otherwise.

## Supplemental Material

sj-docx-1-psp-10.1177_01461672231190719 – Supplemental material for The Improvement Default: People Presume Improvement When Lacking Information Supplemental material, sj-docx-1-psp-10.1177_01461672231190719 for The Improvement Default: People Presume Improvement When Lacking Information by James G. Hillman, Jillian P. Antoun and David J. Hauser in Personality and Social Psychology Bulletin

## Notes

1. See supplemental documents or our OSF.

2. OSF link here: https://osf.io/3nsxm.

3. The preregistration can be found on our OSF: https://osf.io/v63s8.

4. Order of improvement/decline information did not significantly impact perceptions of change in the mixed information group, *t*(94) = 0.10, *p* = .920.

5. Unlike previous studies, participants should have been somewhat familiar with changes in equality from 2003 to 2023, and likely could recall noteworthy events that represented progress (e.g., legalization of gay marriage), causing participants who read decline/stagnation information to still presume improvement overall.

6. All analyses and inferences are the same with behavioral importance as the outcome. These are detailed in the supplementary documents.
