# Supplementary material for: The Improvement Default: People Presume Improvement When Lacking Information
Source: Pers Soc Psychol Bull. 2023 Aug 7;51(1):139–51. doi: 10.1177/01461672231190719 (PMC11616224; doi:10.1177/01461672231190719)
Supplement: sj-docx-1-psp-10.1177_01461672231190719 – Supplemental material for The Improvement Default: People Presume Improvement When Lacking Information [file sj-docx-1-psp-10.1177_01461672231190719.docx]

**Supplemental Documents: Summary**

The supplemental documents for this programme of research are broken down into three sections. In our investigation of this effect, we conducted several studies which could not be included in the full manuscript due to space limitations. For the sake of transparency, we have included five additional studies in Section 1. These studies were either replications of studies in the manuscript or additional studies which sought to explore the improvement default using different methods.

Study 5a explored people’s tendencies to rate general domains as improving or declining, and found participants overwhelmingly reported domains as improving in recent history. Study 5b replicated these results even after selecting domains which had been pretested to demonstrate strong tendencies for decline. Study 6 sought to assess how people’s tendency to assume improvement may bias judgements, in this study we found people reported a desire to recruit the lowest average performing teacher out of three choices if the teacher seemed to have an upward trajectory in their evaluations. In Study7a and 7b we replicated the effects of Study 1a and 1b in the full manuscript, while assessing whether these tendencies emerged due to heuristic thinking. Study 7a is a direct replication of Study 1a with a 6-point rather than 7-point scale. We found that cognitive reflection was not associated to people’s performance on the task in Study 7b. Lastly, in Study 8 we replicated Study 2 using an accountability manipulation to rule out the possibility of heuristic judgements. The accountability manipulation did not affect the replication of the linear effect found in Study 2.

In section 2 we assessed the meta-analytical effect of analyses across studies in both the main document and supplementary. Lastly, in Section 3 we include various additional analyses and data which could not be fit into the main manuscript. We felt these analyses are important for better contextualizing the findings, but do not dramatically alter the conclusions we draw, and so they were not included for the sake of space and brevity.

**Section 1: Study 5a**

To first assess whether people have an intuition that things improve over time, we asked participants to rate whether several domains have improved, remained stable, or declined in recent history. The domains were varied, such that some had exemplars of improvement that could presumably be easily called to mind for most people (e.g., women’s rights) and some were domains where exemplars of improvement would be more difficult to call to mind (e.g., bookbinding). Notably, these ratings did not ask whether participants had improved in these domains over time; rather, they merely asked whether these domains themselves had improved. Participants also rated the degree to which each domain was relevant to themselves in order to affirm that assumptions of improvement exist for non-self relevant domains. We expected that improvement would be endorsed more often than stability or decline and that improvement would not be contingent upon the self-relevance of the domain.

**Method**

***Participants***

A power analysis indicated that 100 participants would be sufficient to find small-medium effects (*d* = .35 and *r* = .30) at 90% power. We recruited 102 undergraduate participants at a midsize Canadian university in southern Ontario. Of our sample 57 (56%) identified as male, 42 (41%) identified as female, and 1 (1%) identified as non-binary. Ages ranged from 23 to 80, (*M* = 38.94, *SD* = 11.58). Participants were remunerated for their time with course credit.

For this and all following studies, power analyses were done using G*Power (Faul, et al., 2009). All data collection and study design were done with the intention to maximize power, accepting a minimum of 90% power for desired effect sizes. Data were analyzed using SPSS statistics software (IBM, 2021) and R (R Core Team, 2020).

***Procedure***

We provided participants a list of 22 domains and asked them to rate whether the given domains had improved or declined in recent history on a 1 *(“Greatly Declined”*) to 7 *(“Greatly Increased”*) scale, with a middle point of 4 (“*No Change*”). We also asked participants whether each domain was relevant to them on a 1 (*“Not relevant”*) to 7 (*“Very Relevant”*) scale. See Table 1 for items.

**Results and Discussion**

To determine whether participants expected improvement, for each item we used a one sample *t* test to test the average improvement rating for that item against the middle, neutral value of 4 (which indicated no change). We also assessed the correlation between each item’s improvement rating and self-relevance to test whether perceptions of improvement were contingent on self-relevance. As such, stronger correlations indicate more shared variance between perceptions of change and self-relevance. See Table 1 for test statistics for all domains.

Of the 22 domains, 20 were identified as improving (90%), one was identified as stable (5%), and one was identified as declining (5%). To assess whether there was an overall tendency to expect improvement, we conducted a chi-square goodness of fit test to determine whether there was a significant difference in frequency of domains being ranked as improvement, stable, or decline, assuming equal proportion of expected values for each domain. This test indicated that participants rated domains as improving significantly more than one might expect from chance χ^2^(2) = 33.15, *p* < .001. Consistent with this, the average perceptions of change across all domains (*M* = 5.17, *SD* = 0.92) was significantly higher than the midpoint *t*(100) = 12.76, *p* < .001, *g* = 1.26, 95% CI [0.98;1.52].

**Table 1**

*Study 5a Items*

| **Domain** | **Mean Ratings** | ***p***  **(t)** | **Mode** | **Effect Size** | **95% CI** | **Self-relevance *r*** | ***p***  **(r)** |
| --- | --- | --- | --- | --- | --- | --- | --- |
| Carpentry technique | 5.25^+^ | < .001 | 6 | 0.81 | 0.58; 1.03 | 0.11 | = .288 |
| Bookbinding | 4.65^+^ | < .001 | 4 | 0.37 | 0.17; 0.57 | 0.27 | =.010 |
| Agriculture | 5.75^+^ | < .001 | 7 | 1.16 | 0.91; 1.41 | -0.04 | =.687 |
| Literacy | 5.96^+^ | < .001 | 7 | 1.52 | 1.22; 1.80 | 0.40 | < .001 |
| Culinary Arts | 5.65^+^ | < .001 | 6 | 1.34 | 1.07; 1.60 | 0.09 | =.399 |
| Chess technique | 4.69^+^ | < .001 | 4 | 0.47 | 0.27; 0.68 | 0.05 | =.637 |
| Music composition | 4.65^+^ | = .001 | 5 | 0.41 | 0.21; 0.61 | 0.23 | =.029 |
| Calligraphy | -3.76^^^- | = .173 | 4 | -0.14- | -0.50; -0.10 | 0.32 | =.002 |
| Piety | -3.56^-^- | = .004 | 4 | -0.29- | -0.49; -0.10 | 0.14 | =.172 |
| Cleanliness | 5.95^+^ | < .001 | 7 | 1.69 | 1.39; 2.00 | 0.42 | < .001 |
| Intellectual debate | 4.83^+^ | < .001 | 6 | 0.51 | 0.30; 0.71 | 0.05 | =.620 |
| Knowledge of History | 5.15^+^ | < .001 | 7 | 0.65 | 0.44; 0.86 | 0.15 | =.167 |
| Understanding of Philosophy | 4.72^+^ | < .001 | 6 | 0.44 | 0.23; 0.64 | 0.39 | < .001 |
| Education | 5.71^+^ | < .001 | 7 | 1.17 | 0.92; 1.43 | 0.26 | =.012 |
| Mastery of spelling and grammar | 4.81^+^ | < .001 | 6 | 0.47 | 0.27; 0.68 | 0.14 | =.192 |
| Metallurgy | 4.98^+^ | < .001 | 4 | 0.59 | 0.38; 0.80 | 0.3 | =.004 |
| Freedom | 5.28^+^ | < .001 | 6 | 0.81 | 0.59; 1.03 | 0.21 | =.048 |
| Happiness | 4.68^+^ | < .001 | 5 | 0.53 | 0.32; 0.73 | 0.25 | =.016 |
| Health and Well-being | 5.74^+^ | < .001 | 7 | 1.12 | 0.87; 1.36 | 0.28 | =.007 |
| Women’s rights | 5.93^+^ | < .001 | 7 | 1.69 | 1.34; 1.99 | 0.27 | =.010 |
| Computer literacy | 6.51^+^ | < .001 | 7 | 2.55 | 2.14; 2.95 | 0.24 | =.024 |
| Diversity | 5.58^+^ | < .001 | 7 | 1.19 | 0.93; 1.44 | 0.43 | < .001 |

*Notes.* Judgements of improvement are indicated by ^+^ judgements of stability are indicated by ^^^ judgements of decline are indicated by ^-^. Effect size is calculated using Hedge’s *g*. *p* values are for *t* score.

As suggested by theories of self-enhancement, self-relevance was moderately correlated with perceptions of improvement, *r*(101) = .41, *p* < .001. This result suggests self-relevance does indeed account for some of the variance in perceptions of change (R^2^ = 16.81). However, only 14 items (64%) had significant correlations between self-relevance and perceptions of improvement. As such, we calculated a chi-square goodness of fit test to determine whether the proportion of items where improvement was correlated versus uncorrelated with self-relevance was greater than what one might expect by chance. Assuming an equal chance that self-relevance was correlated (50%) versus uncorrelated (50%) with improvement, the chi-square test was not significant, χ^2^(1, *N* = 102) = 1.64, *p* = .201 suggesting self-relevance and perceptions of improvement being correlated was not more common than chance. In line with this, several domains had high ratings of improvement with low ratings of self-relevance, such as carpentry technique (*M_change_* = 5.25, *M_self_* = 2.71) or bookbinding (*M_change_* = 4.65, *M_self_* = 2.27). Thus, while self-relevance can certainly spur perceptions of improvement over time, these data suggest that self-relevance is not necessary for perceptions of improvement.

Overall, people presumed that most items that we queried them on have improved in recent history; participants were far more likely to report that domains have improved rather than remained the same or declined. This tendency was often related to the self-relevance; domains that were relevant to the self were often ones where participants saw improvement. However, these judgements were not dependent upon the self-relevance of the domain; domains that were irrelevant to the self (e.g., carpentry technique) were also typically seen as improving. Thus, Study 5a found initial support that a default toward improvement for non-self relevant domains exists.

**Section 1: Study 5b**

While Study 5a demonstrated proof of concept for an improvement default, it is possible that the items we sampled only domains which really do improve (or, at least, ones that most people generally believe improved). Study 5b addresses this concern. We sought to replicate the pattern of results in Study 5b; however, we aimed to test beliefs of improvement for domains which did *not* exhibit recent improvement and, in fact, are generally believed to have recently declined. This extension would eliminate any potential sampling default and provide evidence of a robust improvement default.

To assemble a list of domains which people think have declined, we first ran a pre-test which asked undergraduate students to nominate domains that they felt have declined in recent history. We compiled nominations into semantically-similar terms to create a list of the twenty most common domains reported as declining by undergraduate students. Then, in a design similar to Study 5a, we asked a new group of undergraduate participants to rate whether these 20 domains had declined, remained stable, or improved.

**Method**

***Pretest Procedure***

To first generate a set of domains for which decline should be expected, we recruited 181 undergraduate participants at a midsize Canadian university in southern Ontario. Of these participants, 161 (89%) identified as female, 16 (9%) identified as male, 2 (1%) identified as non-binary, and 2 (1%) declined to provide any gender information. Ages ranged from 18 to 40 (*M* = 19.76, *SD* = 5.26).

The data were collected digitally over Qualtrics. Participants were shown the following prompt: “*We are generally interested in learning how people think of change over time. In particular, we want to know what people think is declining over time. In the space below please identify 5 to 10 domains which you feel have been declining over time in recent history.*” Participants were given a text box to provide responses.

***Pretest Results***

Participants provided hundreds of unique responses. However, many were conceptually the same. For instance, while several participants provided the domain “*well-being*” others provided the domain “*mental well-being*” or “*mental wellness*” or “*mental state.*” As such, we decided that thematically-similar domains such as these should be grouped into superordinate categories such as “*mental health.*” A research assistant who was blind to the research hypotheses categorized participant responses based on thematic similarity, yielding 20 domains (see Table 2 for the complete list). Further, the kinds of domains participants identified were numerous and often very idiosyncratic, suggesting a high degree of individual variance in perceptions of decline.

***Main Study Procedure***

Because of their methodological similarities, we assumed similar power criteria as Study 5a. We recruited 113 undergraduate participants at the same university as the pretest. Individuals who participated in the pretest could not participate in this study. Of our sample 95 (84%) identified as female and 16 (14%) identified as male, 2 (2%) participants identified as non-binary, or declined to provide gender information. Ages ranged from 17 to 49, (*M* = 18.89, *SD* = 4.12). Participants were remunerated for their time with course credit.

We provided participants a list of the twenty domains nominated as declining in pretesting. As with Study 5a we asked participants to rate whether the given domains had improved or declined in recent history on a 1 *(“Greatly Declined”*) to 7 *(“Greatly Increased”*) scale, with a middle point of 4 (“*No Change*”).

**Main Study Results and Discussion**

To determine whether participants reported improvement on average, we used one sample *t* tests to test against the middle, neutral value of 4 (which indicated no change). See Table 2 for these values.

**Table 2**

*Study 5b Results*

| **Domain** | **Mean**  **Ratings** | ***p*** | **Mode** | **Effect Size** | **95% CI** |
| --- | --- | --- | --- | --- | --- |
| Literacy | 4.73^+^ | < .001 | 6.00 | 0.49 | 0.29; 0.68 |
| Freedom | 4.88^+^ | < .001 | 5.00 | 0.58 | 0.38; 0.78 |
| Nature | 3.20^-^- | < .001 | 2.00 | -0.47- | -0.67; -0.28 |
| Equality | 5.27^+^ | < .001 | 6.00 | 1.14 | 0.90; 1.38 |
| Mental Health | 3.47^-^- | =.005 | 1.00 | -0.27- | -0.45; -0.08 |
| Education | 1.34^+^ | < .001 | 6.00 | 0.89 | 0.68; 1.11 |
| Relationships | 4.30^+^ | =.042 | 5.00 | 0.19 | 0.01; 0.38 |
| Socialization | 4.49^+^ | =.009 | 6.00 | 0.25 | 0.06; 0.44 |
| Altruism | 4.21^+^ | =.042 | 4.00 | 0.20 | 0.01; 0.38 |
| Art | 4.21^^^ | =.136 | 4.00 | 0.14 | -0.04; 0.33- |
| Communication | 5.17^+^ | < .001 | 6.00 | 0.75 | 0.54; 0.95 |
| Integrity | 4.20^^^ | =.119 | 4.00 | 0.15 | -0.04; 0.33- |
| Health | 4.64^+^ | < .001 | 6.00 | 0.37 | 0.18; 0.56 |
| Love | 4.35^+^ | =.017 | 4.00 | 0.23 | 0.04; 0.42 |
| Happiness | 3.81^^^- | =.158 | 3.00 | -0.13- | -0.32; 0.05- |
| Respect | 4.55^+^ | < .001 | 6.00 | 0.39 | 0.20; 0.58 |
| Privacy | 3.14^-^- | < .001 | 2.00 | -0.52- | -0.71; -0.32 |
| Connections | 4.58^+^ | < .001 | 6.00 | 0.33 | 0.14; 0.52 |
| Religiosity | 3.42^-^- | < .001 | 4.00 | -0.40- | -0.59; -0.21 |
| Empathy | 4.53^+^ | < .001 | 5.00 | 0.36 | 0.17; 0.55 |

*Notes.* Judgements of improvement are indicated by ^+^ judgements of stability are indicated by ^^^ judgements of decline are indicated by ^-^. Effect size is calculated using Hedge’s *g*. *p* values are for t score.

The results of the one sample *t* tests indicated participants reported that 13 (65%) domains had improved in recent history, 3 (15%) had stayed the same, and 4 (20%) had declined. That is, even when we sampled domains that participants’ peers had often and explicitly identified as having declined in recent years, participants, on average, felt that nearly three times more domains improved than stayed the same or declined (individually). As with Study 5a we used a chi-square goodness of fit test to assess whether domains were seen as improving (rather than staying the same or declining) at a significantly higher rate than chance. We assumed equal proportion of expected values (33% for each trajectory). This test indicated that participants rated domains as improving significantly more than one might expect from chance χ^2^(2, *N* = 113) = 9.19, *p* = .010. As with Study 5a, the average perception of change across all domains (*M* = 4.33, *SD* = 0.81) was significantly higher than the midpoint *t*(112) = 4.30, *p* < .001, *g* = 0.40, 95% CI [0.21;0.60].

People seem to have a robust presumption that most things have gotten better even in areas their peers nominated as declining. A pre-test collected the twenty most frequently mentioned domains that have declined in recent history. However, a second group of participants, facing the question of whether those same domains had improved, remained stable, or declined in recent history, reported that these domains had, on average, improved in recent history. Thus, the mental processes involved in nominating domains that have declined and evaluating whether a domain has improved versus declined appear to drastically differ. This tendency may occur because of the way people bring to mind exemplars, for instance, if one is asked “where can you buy pasta sauce” they may not readily call to mind ‘convenience store’ but if they are asked ‘how likely is it you’d find pasta sauce in a convenience store,’ they may agree it’s at least a little likely. Overall, this study demonstrates that when evaluating a domain with no other information, people’s presumptions skew toward improvement over time.

**Section 1: Study 6**

While the previous studies demonstrated that the improvement default impacts judgments of whether things change over time, Study 6 examined a potential implication of this tendency. The improvement default may serve as an expectation against which people evaluate things that change over time. Judgment targets that improve over time may be expected or appealing, while targets that remain stable or decline may fail to meet expectations. In particular, in light of research on comparative effects of positive and negative information (Baumeister et al., 2001; O'Brien & Klein, 2017; Rozin & Royzman, 2001), one may view a good option as less desirable because it declined or even stayed the same, while viewing a worse option as better simply because it bears the resemblance of improvement. This expectation could bias evaluations of those targets by carrying more weight in decision-making than other diagnostic information, such as overall performance across time.

Participants evaluated three course instructors, choosing which of three instructors they would recommend be hired at a university. Each instructor’s teaching ratings over successive terms were visually graphed, which gave the impression that one instructor’s ratings improved, another’s remained stable, and another’s declined. However, the prompt described clear confounds which made rating trajectory an unreliable indicator of future performance (e.g., each candidate taught a different course each term). Additionally, the instructor whose ratings declined had the *highest average* performance across terms, followed by the instructor whose ratings remained stable, followed by the instructor whose ratings improved. Thus, if one were to consult average teaching scores, the best option would be the seemingly declining one, then the stable one, then the seemingly improving one. We hypothesized that the teacher whose ratings were in a seemingly upward trajectory (i.e., had a positive slope) would be ranked as the most desirable hire even though they had the lowest average ratings.

**Method**

***Participants***

Power analysis indicated that to detect an average effect size (φ = 0.30) we would need 141 participants to reach 90% power. As such, we recruited 147 participants from Amazon’s Mechanical Turk. To ensure data quality, we used Cloud Research approved participants (Hauser et al., 2022). Of the 147 participants, 86 (58%) identified as male, 60 (41%) identified as female, 1 participant identified as non-binary or did not disclose their preferred gender identity (1%). Participant ages ranged from 21 to 69 (M = 37.07, SD = 11.50). Participants were compensated for their time with $0.15 per estimated minute of participation.

***Procedure***

Participants were recruited for a task on person impressions and were asked to evaluate potential teaching candidates for a university. Participants viewed a graph with three lines indicating the teaching evaluations of the three candidates. Each candidate had a rating for three successive terms (see Figure 1). Participants ranked candidates in order of preference (first, second, third).

We specified that during each term, each candidate taught only one course, and all the courses were different. The figure was designed such that Teacher A’s ratings improved over time, Teacher B’s ratings remained stable over time, and Teacher C’s ratings declined over time. However, the average evaluation across terms was 4.33 for Teacher A, 5 for Teacher B, and 5.33 for Teacher C. These ratings were selected to give a sizable mean difference among groups, without making the difference immediately obvious. Also, Teacher A had clearly worse ratings than Teacher B and C in two of the three terms. Thus, several diagnostic criteria (average rating and performance each term) indicate that Teacher A was the worst candidate.

**Figure 1**

*Study 6 Stimuli*

**Results and Discussion**

We computed a chi-square test to determine whether selections for both first and second choice of teacher exceeded amounts one might expect from chance. For first choice, the chi-square test was significant, χ^2^ (2, *N* = 147) = 84.59, *p* < .001, φ = 0.76. As shown in Table 3 and as hypothesized, most participants (66%) ranked Teacher A, whose trajectory fit the improvement default but whose ratings were objectively worse on average, as their first choice. Only 29% of participants chose Teacher B and 5% chose Teacher C as their first choice. Thus, the improvement default serves as a standard of comparison for things that change over time; improvement over time is expected and carries more weight in evaluations than other objectively more diagnostic criteria.

| **Table 3**  *Study 6 Results* |  |  |  |
| --- | --- | --- | --- |
| **Choice** | **Teacher** | **N of participants** | **% of participants** |
| First | A | 97 | 66% |
|  | B | 43 | 29% |
|  | C | 7 | 5% |
| Second | A | 34 | 23% |
|  | B | 102 | 70% |
|  | C | 11 | 7% |

We repeated the same chi-square test for second choice, which was also significant, χ^2^ (2, *N* = 147) = 91.39, *p* < .001, φ = 0.79. As shown in Table 3, 70% of participants preferred Teacher B as their second choice. Only 23% chose Teacher A and only 7% chose Teacher C as their second choice.

Despite having the lowest average performance and the objectively worst performance in most terms, the teacher who *seems* to be improving over time was preferred much more than the other two teachers. It was made clear to participants that each course taught in each term was different, so they were made aware of the potential for confounding factors that could bias trajectories (e.g., Teacher A taught a course that historically receives more favourable ratings in their final term, or teaching evaluation criteria changed in the final term, or random noise, etc.). Regardless, participants overwhelmingly preferred the teacher with the lowest average scores and an appearance of improvement. These results demonstrate how people’s default tendency to presume improvement may lead them to prefer things which seem to be growing and cause them to overlook more diagnostic criteria.

**Section 1: Study 7a**

A theme across narratives in North America is one of improvement. The ubiquity of this theme may foster a default belief that the trajectories of most domains bend toward improvement. As such, when evaluating whether a domain improves/declines over time, people may have the default and highly accessible intuition of improvement.

In Study 1a, participants evaluated whether several domains had improved, remained stable, or declined over historical periods [e.g., “Happiness for Romans in Rome from 210 AD to 410 AD”]. Domains were ones associated with historical decline; for instance, the years between 210 AD to 410 AD exhibited decline for the Roman Empire as Romans suffered from invasion, disease, and famine, culminating in the three-day long sacking of Rome in 410 made possible by mass starvation (Heather, 2006). Half of the items contained additional information diagnostic of decline [e.g., “Happiness for Romans in Rome from 210 AD to 410 AD (the decline and sacking of Rome)”] while the other half contained none. This design removes both the participants’ selves and futures from the evaluation. As such, there should be no motivation stemming from self-enhancement or optimism to perceive these time periods as improving more than staying stable or declining.

We expected the improvement default to manifest only for ambiguous judgments (i.e., judgements lacking diagnostic information; Kruger et al., 2004; Todorov et al., 2002). Thus, we hypothesized that participants would (erroneously) expect improvement for items containing no additional diagnostic information, and participants would (accurately) not expect improvement for items containing additional diagnostic information.

**Method**

***Participants***

Power analyses indicated 230 participants would be sufficient to reach 90% power for a small within-between ANOVA interaction (η^2^ = 0.02), assuming minimal correlation (*r* = .10) between measures. As such, we recruited 244 participants from a midsized Canadian university in southern Ontario. We removed 17 participants for failing to respond to our comprehension check. Of the remaining 227, 172 participants identified as female (77%) and 52 identified as male (23%). The mean age of the sample was 19 years old (*SD* = 3.14).

***Procedure***

Participants viewed eight timeframes in which historical decline occurred (e.g., Rome from 210 to 410), and evaluated a relevant domain for each timeframe (e.g., happiness for Romans). Half of the items contained diagnostic information in the form of a concise “hint” as to what historically happened in that timeframe (e.g., “the decline and sacking of Rome”) while the other half did not.

We split the eight items into two blocks, each with four items (Block A and Block B). We used Block (A vs B) as a between-subjects variable determining which items contained diagnostic hints and which items did not. That is, for participants assigned to hinted item Block A (B), items in Block A (B) contained hints while items in Block B (A) did not. As such, each participant was exposed to half of the timeframes with hints and half with no hints (creating a within-subjects factor of hint), and which particular items had hints was a between-subjects blocking factor. For each item, participants rated whether the given domain had improved or declined during the given time frame on a 1 (*“Greatly Declined”*) to 6 (*“Greatly Improved”*) scale.

To avoid participants feeling obligated to use the entire scale, participants also completed two filler items where historical improvement was likely to have occurred (e.g., health in Europe from 1941 to 2011). Responses to these items were not analyzed.

**Results and Discussion**

We used a 2 between (hinted item block: A vs B) by 2 within (information: hint vs no hint) mixed model ANOVA on ratings of change. As hypothesized, the within-subjects main effect of information was significant *F*(1,224) = 62.72, *p* < .001, η^2^ = .22, 95% CI [.13;.31]. The between subjects main effect of hinted item block was not significant *F*(1,224) = 1.23, *p* = .268, η^2^ = .01, 95% CI [.00;.04]. Unexpectedly, there was a significant interaction between information and hinted item block *F*(1,224) = 4.76, *p* = .030, η^2^ = .02, 95% CI [.00;.07].

Our critical hypotheses, however, concerned the effect of diagnostic information (e.g., hints). We performed two pairwise t-tests assessing the effect of information at each level of hinted item block. As shown in Figure 2, for participants whose Block A contained hinted items, there was a significant difference in ratings of change between items with hints and items without hints, *t*(112) = 7.43, *p* < .001, *g* = .70, 95% CI [.49;.90]. Similarly, for participants whose Block B contained hinted items, there was also a significant difference in ratings of change between items with hints and items without hints, *t*(112) = 3.91, *p* < .001, *g* = .37, 95% CI [0.18;0.56]. Thus, the interaction suggests that the effect of hint is significant in both blocks but is stronger for one than the other.

We also assessed whether average ratings were significantly different from the theoretical midpoint of the scale (3.5). In line with the improvement default, the items without hints (*M* = 3.64, *SD* = 0.87) were rated as generally improving (i.e., significantly higher than the midpoint), *t*(225) = 2.49, *p* = .014, *g* = .17, 95% CI [0.03;0.30]. As suggested by ambiguity as a moderator of the improvement default, the sections with hints (*M* = 3.16, *SD* = 0.99) were rated as generally declining (i.e., significantly lower than the midpoint), *t*(225) = -5.24, *p* < .001, *g* = .35, 95% CI [0.21;0.48].

**Figure 2**

*Perceptions of Change by Block and Hint*

*Note.* Values greater than 3.5 indicate improvement, values less than 3.5 indicate decline. Error bars indicate standard error.

As hypothesized, the improvement default operates according to the rules of intuitively-driven biases (Kruger et al., 2004; Todorov et al., 2002) and social cognitive principals of knowledge accessibility (Anderson, 1971; Higgins, 1996). When evaluating change in historical periods associated with decline, participants rated the domains as improving when diagnostic information was lacking. This result suggests that, when facing ambiguous judgments, people default to presume improvement and endorse it as their response. However, when diagnostic information about decline was available, participants eschewed the improvement default and rated the domains as declining. Notably, evaluation targets were retrospective appraisals of foreign countries, meaning that neither optimism nor self-enhancement explains these findings. Instead, beyond previously documented effects, these findings suggest that people have a default to presume improvement over time. This default leads people to incorrectly report improvement in domains where decline had occurred. Like other intuitions, improvement is endorsed when lacking diagnostic information and abandoned when better information is readily available.

**Section 1: Study 7b**

Our previous studies provided some evidence suggesting that the improvement default functions as an intuition in which improvement is presumed when lacking sufficient information. To assess whether this functioned like a heuristic (i.e., employed when people are not thinking hard but abandoned when they do), this study explored whether situational differences in intuitive versus reflective thinking were related to people’s presumption of improvement over time. Replicating the methods of our prior studies, participants indicated if various timespans reflected improvement, no change, or decline. For some participants, hints provided information suggesting that decline occurred during the timespan, whereas for other participants, no hints were provided. In addition, participants completed an updated Cognitive Reflection Test (CRT-2). We expected to replicate the finding that when no hints are provided, participants presume improvement over time. Additionally, if the improvement default stems from heuristic processing, we hypothesized that participants displaying less cognitive reflection would presume greater improvement over time than would participants displaying high cognitive reflection.

**Method**

***Participants***

Participants (*N* = 318) were recruited from Amazon’s Mechanical Turk. Each participant was compensated with $0.15 USD per pre-tested minute of completing the survey for a total of $0.45USD. Although 318 participants began the survey, 19 dropped out prior to completing, and 29 failed the attention check, leaving the final sample total of 270. In this sample, 151 identified as male, 113 identified as female, 2 identified as non-binary, and 3 preferred not to say. The sample had a mean age of 39.93 years.

***Materials and Procedure***

The survey was presented to participants online via Qualtrics. After reading the letter of information and providing consent, participants proceeded to the survey. The survey was identical to the original study; the only modification was the additional measure of the CRT-2 as the final task.

The survey began with participants evaluating the degree to which change in a domain occurred during a historical time period. All items were identical to the ones used in Study 7a. Participants rated 10 of these items on a 7-point scale where 1 represented *Greatly Declined*, 4 represented *No Change*, and 7 represented *Greatly Improved*. Diagnostic information about decline during the historical time period (i.e., one hint per item) was randomly assigned between-subjects. That is, half of the participants received hints that indicated that the historical time periods were associated with decline, whereas the other half of the participants received no such hints. Although the eight periods of interest were times of decline for the given domain, two additional items that referred to periods of improvement were included as foils but were not analyzed. Item order was randomized.

After completing the ratings, participants completed the CRT-2 (Thomson & Oppenheimer, 2016). The CRT tests one’s ability to resist an intuitive yet incorrect answer to different problems (Frederick, 2005). Reflective thinking is necessary for overriding the intuitive answers that are prompted by the CRT items (Evans & Stanovich, 2013). For instance, this updated test included items such as, *“*A farmer had 15 sheep and all but 8 died. How many are left?” The intuitive response that individuals often think of is 7. However, this intuitive answer is incorrect. The correct response would be 8. Higher scores on the CRT-2 indicate a higher propensity to resist intuitive answers and use reflective thinking to continue searching for the correct response. The CRT-2 was developed because participant pools have been heavily exposed to the original CRT items, raising concerns of the original measure’s validity (Thomson & Oppenheimer, 2016). To measure situational cognitive reflection, the four CRT-2 questions were presented to participants on the same survey page, and participants typed responses in a text-entry box. Finally, participants filled out demographic information, read the debriefing form, and accessed their compensation through a dynamic link at the end of the survey.

**Results**

The results of Study 7a found an effect of hint such that non-hinted items were perceived as having more improvement than hinted items. Additionally, participants in previous studies rated non-hinted items on average as significantly above the “no change” midpoint of the scale, indicating that they presumed improvement in those domains over the given timespan. In the present analyses, we assessed if these effects were moderated by CRT-2 scores.

To form an index of perceptions of improvement, we averaged participants’ change ratings for the eight items, Ω = 0.82 (hint), Ω = 0.47 (no hint). We submitted these perceptions of improvement to a moderated regression, with mean-centered CRT scores, a dummy-coded Hint variable (0 = Hint, 1 = No Hint), and their two-way interaction entered as predictors. Replicating our prior research, the simple effect of hint at the average of CRT scores was significant, *b* = 1.013, *t*(265) = 3.139, *p* = .001. Participants receiving hints rated the change over time as significantly lower (*M* = 3.88, *SD* = 1.26) than participants receiving no hints (*M* = 5.12, *SD* = 0.88). Thus, the previously observed effect of hint was replicated. The Hint X CRT interaction was not significant, *b* = 0.084, *t*(265) = 0.736, *p* = .462, so we did not find evidence of significant moderation of the hint effect by CRT. To assess if the effect of hint reflected an improvement default, we conducted a one-sample t test for both the hints group and the no hints group to see if their perceptions of improvement were significantly different from the “no change” midpoint of the scale (i.e., a rating of 4). The “hints” group (*M* = 3.881, *SD* = 1.261) did not perceive domains as significantly improving or declining, *t*(133) = 1.090, *p* = .139. However, the no hints group (*M* = 5.117, *SD* = 0.885) perceived domains as significantly improving, *t*(136) = 14.766, *p* < .001. Therefore, on average, participants that received diagnostic information about times of decline did not expect improvement, but participants without diagnostic information did expect improvement. This study suggests that the improvement default is not just people not thinking, but rather it seems more like a product of a lack of information.

**Section 1: Study 8**

In Study 8, we continued to explore the mechanism underlying the improvement default by examining whether a manipulation of intuitive versus reflective thinking would affect people’s presumption of improvement over time. Accountability has been shown to reliably cause participants to think more effortfully in making judgements (Tetlock, 1983). Thus, we randomly assigned participants to feel accountable or not for their judgment. Then, participants placed events of varying negativity and positivity in temporal order. We expected to replicate prior results such that participants, on average, should order events in a sequence that indicates increasing positivity over time. If intuitive thinking plays a role in the improvement default, then we hypothesized that participants in the accountability condition would not order events in increasing positivity over time, but participants in the control condition would.

**Method**

***Participants***

Participants (*N* = 600) were recruited from Amazon’s Mechanical Turk. Each participant was compensated with $0.15 USD per pre-tested minute of completing the survey. Only 512 participants completed the study in its entirety. Data pre-processing consisted of excluding 11 participants that failed the attention check, leaving a final sample of *N* = 501. In our sample, 266 participants identified as male, 229 as female, 3 as non-binary, and 3 preferred not to say. The sample had a mean age of 23.248 years.

***Materials and Procedure***

The survey was presented to participants online via Qualtrics. After reading the letter of information and providing consent, participants proceeded to the survey. First, all participants received an introduction to the process accountability manipulation. Participants were told that they would be making a judgement and that they would need to explain what they thought about while reading the scenario and describe why they made the judgement they did. Then, participants completed a gambler’s fallacy decision task in which they indicated how many times they believed an individual had rolled a set of dice based on a short text (Oppenheimer & Monin, 2009). They also provide their rationale in a large text box. Because accountability manipulations make participants do difficult tasks that are atypical for the MTurk platform, we exposed all participants to an accountability manipulation at the onset to encourage participants who were not willing to undergo a difficult task to drop out prior to random assignment to groups. The aim was to prevent complications due to differential dropout between accountability vs no accountability conditions (Zhou & Fishbach, 2016).

Following this initial task, participants were randomly assigned between subjects to either the accountability or no accountability manipulation (Brtek & Motowidlo, 2002; Pitesa & Thau, 2013; Tetlock, 1983). Participants in the accountability condition were prompted with accountability instructions once again before completing the experimental task. Alternatively, participants in the control group were given instructions informing them that their responses in the upcoming task were completely anonymous and confidential, and that an explanation of their judgment would not be requested.

Following these instructions, the survey replicated the design of Study 2 demonstrating the improvement default. That is, participants learned about an individual named Denise who had five events happen to her over the course of five weeks. Two of the events were positively valenced (e.g., “Denise found a $50 bill on the ground”), two were negative (e.g., “A driver in the parking lot yelled at Denise”), and one was neutral (e.g., “Denise left a note in her car to pick up the mail after work”). Participants were to put the events in the order that they thought the events occurred during the five-week span (e.g., week 1 to week 5). Then, participants rated how positive they felt that each event was for Denise on a scale of 1 to 7, where 1 represented *Very Negative* and 7 represented *Very Positive*. Afterwards, participants read the debriefing form and accessed their compensation through a dynamic link at the end of the survey.

**Results**

Our previous research found linear and quadratic effects of event order on positivity ratings of events. For the linear effect, participants expected events to get more positive as the order progressed (e.g., events occurring in later weeks being more positive than events occurring in earlier weeks). For the quadratic effect, participants expected initial events (week 1) to be neutral, events occurring in the middle of the time period (week 3) to be negative, and events occurring at the end of the time period (week 5) to be the most positive. In this study, we assessed if these linear and quadratic effects of event order would replicate and if the effect would be moderated by accountability.

A 5 within (event order: week 1, week 2, week 3, week 4, week 5) X 2 between (accountability: accountable, not accountable) mixed-design ANOVA was conducted on event positivity ratings. To assess replication of prior effects, we also tested two planned polynomial contrasts for the effect of event order: a linear contrast and a quadratic contrast.

Replicating prior research, the main effect of event order was significant, *F*(4, 495) = 14.982, *p* < .001. As expected, the linear contrast for event order was significant, *F*(1, 499) = 11.555, *p* < .001, but the quadratic contrast was not significant,  *F*(1, 499) = 10.504, *p* = .195. As shown in Table 4, participants presumed improvement over the course of five weeks, rating events occurring in successive weeks as improving over time. Thus, the predicted improvement default replicated.

However, the effect of event order was not moderated by accountability, *F*(4, 495) = 1.143, *p* = .334, nor was the main effect of accountability significant, *F*(1, 499) = 1.791, *p* = .181. Therefore, in this paradigm, the improvement default was not significantly attenuated when people were held accountable for their judgment, suggesting that the effect may not depend on heuristic processing. Taken into consideration with the results of Study 7b, it is plausible that the improvement default involves both heuristic and systematic processing, which is why the present research was unable to detect evidence of a heuristic-based mechanism. This further suggests the improvement default is not solely a heuristic used when individuals are not thinking hard, but an intuition employed when lacking information.

**Table 4**

*Mean (SD) Event Valence as a Function of Event Order in Study 8*

| Event order | Mean valence (SD) |
| --- | --- |
| Week 1 | 3.940 (.158) |
| Week 2 | 3.477 (.148) |
| Week 3 | 4.664 (.140) |
| Week 4 | 3.885 (.153) |
| Week 5 | 4.643 (.154) |

**Section 2: Meta Analyses of Studies**

Because the methodology was similar across several studies, we conducted a meta-analysis with a random effects model using maximum likelihood estimations. We computed a meta-analytic effect for comparisons between Information/No Information conditions, as well as comparison of each condition to the middle point of the given scale.

***Information and No Information Comparisons***


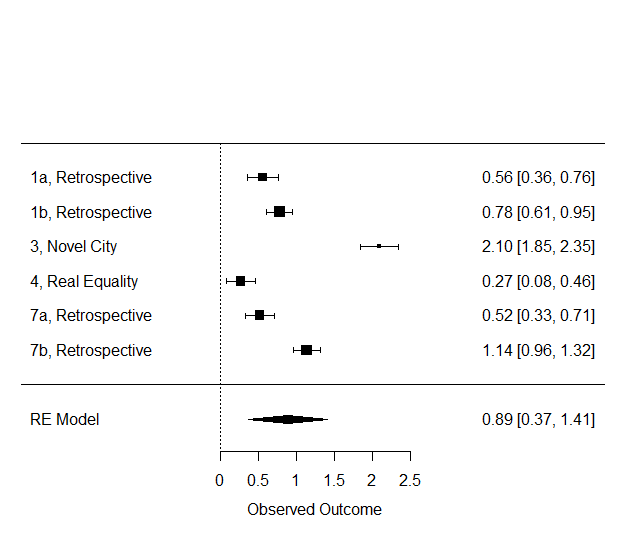
 We expected across methodologies, individuals with relevant information would have lower (more declining) ratings of change than those with no information. The meta-analytical effect supported this, as it was significantly larger than zero *g* = 0.89, 95% CI [0.37; 1.41]. Figure 3 demonstrates this effect.

**Figure 3**

*Comparison of Information and No Information Conditions*

***No Information and Midpoint Comparisons***

We expected across methodologies, individuals with no additional information would have mean ratings of change higher than the midpoint (i.e., reporting improvement). The meta-analytical effect supported this, as it was significantly larger than zero *g* = 0.63, 95% CI
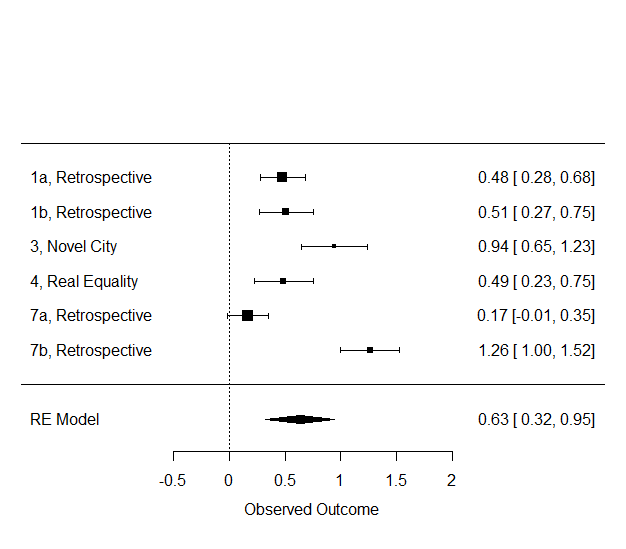
[0.32;0.95]. Figure 4 demonstrates this difference.

**Figure 4**

*Comparison of No Information Conditions and Midpoint*

***Information and Midpoint Comparisons***

We expected across methodologies, individuals with additional information would have ratings lower than the midpoint (i.e., reporting decline). The meta-analytical effect supported this, as it was significantly larger than zero *g* = -0.41, 95% CI [-0.77;-0.03]. Figure 5 demonstrates this difference.


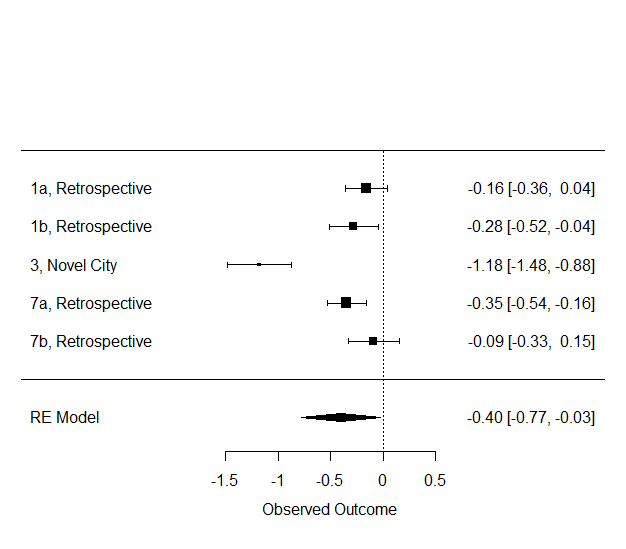
**Figure 5**

*Comparison of Information Conditions and Midpoint*

**Section 3: Study 1a**

***Scale Information***

We assessed the reliability of items in each block. The internal reliability was mediocre for both block A (α = .62) and B (α = .64). Two main factors probably negatively impact the reliability of these items. First, some items have hints and others do not. This could (and according to our hypothesis should) make people think differently about the items. Secondly, there are ‘correct’ answers for all these items, which require some degree of familiarity or knowledge, even without hints. People’s knowledge of history will largely be idiosyncratic, and so we expect relatively low correlations between domains. This expectation is supported by relatively low inter-item correlations, suggesting people generally have idiosyncratic responses between items. See Table 5.

**Table 5**

*Internal Reliability of Items*

| **Block A (No Hints)**  Item 1 (Item 2: -.05, Item 3: .02, Item 4: .03)  Item 2 (Item 1: -.05, Item 3: .16, Item 4: .36*)  Item 3 (Item 1: .02, Item 2: .16, Item 4: .25*)  Item 4 (Item 1: .03, Item 2: .36*, Item 3: .25*) | **Block B (Hints)**  Item 1 (Item 2: .15, Item 3: .24, Item 4: .15)  Item 2 (Item 1: .15, Item 3: .27*, Item 4: .56*)  Item 3 (Item 1: .24*, Item 2: .27*, Item 4: .08)  Item 4 (Item 1: .15, Item 2: .56*, Item 3: .08) |
| --- | --- |
| **Block A (Hints)**  Item 1 (Item 2: .13, Item 3: .41*, Item 4: .13)  Item 2 (Item 1: .13, Item 3: -.21*, Item 4: .10)  Item 3 (Item 1: .41*, Item 2: -.21*, Item 4: .27*)  Item 4 (Item 1: .21, Item 2: .10, Item 3: .27*) | **Block B (No Hints)**  Item 1 (Item 2: .15, Item 3: .22*, Item 4: .27*)  Item 2 (Item 1: .15, Item 3: .13, Item 4: .15)  Item 3 (Item 1: .22*, Item 2: .13, Item 4: .37*)  Item 4 (Item 1: .27*, Item 2: .14, Item 3: .37*) |

We also assessed the correlation between hints and no hints groups and the filler items. The No Hint group was moderately correlated with filler 5 (*r =* .37*, p <* .001) and 10 (*r =* .22*, p =* .011). The hint group was not correlated with filler 5 (*r =* .07*, p =* .390) but was with 10 (*r =* .35*, p <* .001).

***Estimated Marginal Means***

As per our analysis in the main document, see Table 6 for estimated marginal means and standard errors.

**Table 6**

*Estimated Marginal Means and Standard Errors of Blocks*

|  | **Block A** | **Block B** |
| --- | --- | --- |
| Hint | 4.15 (.11) | 3.51 (.11) |
| No Hint | 4.48 (.10) | 4.45 (.10) |

The difference between Block A and Block B on Hints was significant (*p* < .001), while the difference between Block A and Block B on No Hints was not (*p* = .838). The difference between Hints and No Hints in Block A was significant (*p* = .005), and the difference between Hints and No Hints in Block B was significant (*p* < .001).

***Number of Participants Indicating Improvement***

Because mean scores can be biased by a relatively low concentration of very extreme scores, we also compared the relative percentages of participants who selected *any* decline or improvement on average. In line with our hypotheses, we expected that participants should endorse improvement more often than decline/stability when given no information, but less often when given information. See Table 7 for details.

**Table 7**

*Count of Participants Indicating Improvement*

|  | **Decline** | **Stability** | **Improve** | **Totals** |
| --- | --- | --- | --- | --- |
| Hint | 99 (53%) | 18 (10%) | 69 (37%) | 186 |
| No Hint | 46 (25%) | 25 (13%) | 115 (62%) | 186 |
| Totals | 145 | 34 | 184 | 372 |

There was a significant difference based on group with a chi-square test, χ^2^ = 32.01, *p* < .001. When given information, people endorsing decline moved from 25% to 53% and people expecting improvement went from 37% to 62%. This result is consistent with the main manuscript, suggesting that with no hints people are far more likely to report improvement and less likely to report decline or stability. However, when given relevant information (about decline), people are more likely to report decline or stability over improvement.

**Section 3: Study 1b**

Part of the low inter-item correlations and reliability observed in Study 1 could be due to the within-subjects method. Both reliability for Hints (α = .74) and No Hints (α = .75) groups were better within the acceptable range better than Study 1a. The inter-item correlations were also notably better (see Table 8 and 9, note that items 5 and 10 were the filler items, which were removed), suggesting the inconsistencies in Study 1a may have been due to the mixed model design.

**Table 8**

*Inter-item Correlations for No Hints Group*

|  | | | | | | | | |
| --- | --- | --- | --- | --- | --- | --- | --- | --- |
|  |  | Item 2 | Item 3 | Item 4 | Item6 | Item 7 | Item 8 | Item 9 |
| Item 1 | *R* | .196* | .241** | .176* | .203* | .235** | 0.132 | .260** |
|  | *p* | 0.022 | 0.005 | 0.040 | 0.018 | 0.006 | 0.126 | 0.002 |
| Item 2 | *R* | 1 | .276** | .314** | .335** | .263** | .259** | 0.137 |
|  | *p* |  | 0.001 | 0.000 | 0.000 | 0.002 | 0.002 | 0.111 |
| Item 3 | *R* |  | 1 | .183* | .315** | .302** | .407** | .357** |
|  | *p* |  |  | 0.033 | 0.000 | 0.000 | 0.000 | 0.000 |
| Item 4 | *R* |  |  | 1 | .515** | .250** | .296** | .229** |
|  | *p* |  |  |  | 0.000 | 0.003 | 0.000 | 0.007 |
| Item 6 | *R* |  |  |  | 1 | .269** | .215* | .256** |
|  | *p* |  |  |  |  | 0.002 | 0.012 | 0.003 |
| Item 7 | *R* |  |  |  |  | 1 | .229** | .314** |
|  | *p* |  |  |  |  |  | 0.008 | 0.000 |
| Item 8 | *R* |  |  |  |  |  | 1 | .255** |
|  | *p* |  |  |  |  |  |  | 0.003 |

**Table 9**

*Inter-item Correlations for Hints Group*

|  | | | | | | | | |
| --- | --- | --- | --- | --- | --- | --- | --- | --- |
|  |  | Item 2 | Item 3 | Item 4 | Item6 | Item 7 | Item 8 | Item 9 |
| Item 1 | *R* | .303** | .176* | .184* | 0.001 | .270** | 0.152 | .199* |
|  | *p* | 0.000 | 0.038 | 0.030 | 0.993 | 0.001 | 0.075 | 0.019 |
| Item 2 | *R* | 1 | .301** | .545** | .420** | .266** | .406** | .229** |
|  | *p* |  | 0.000 | 0.000 | 0.000 | 0.002 | 0.000 | 0.007 |
| Item 3 | *R* |  | 1 | .304** | .381** | .303** | 0.156 | .211* |
|  | *p* |  |  | 0.000 | 0.000 | 0.000 | 0.068 | 0.013 |
| Item 4 | *R* |  |  | 1 | .397** | .196* | .362** | .297** |
|  | *p* |  |  |  | 0.000 | 0.021 | 0.000 | 0.000 |
| Item 6 | *R* |  |  |  | 1 | .423** | .293** | .205* |
|  | *p* |  |  |  |  | 0.000 | 0.000 | 0.016 |
| Item 7 | *R* |  |  |  |  | 1 | .187* | .178* |
|  | *p* |  |  |  |  |  | 0.028 | 0.036 |
| Item 8 | *R* |  |  |  |  |  | 1 | .295** |
|  | *p* |  |  |  |  |  |  | 0.000 |

We also assessed the correlation between hints and no hints within each block, as well as the filler items within each block. For block A, hint and no hint groups were moderately correlated *r =* .53*, p* < .001. The first filler item was not correlated to the Hint (*r =* .03*, p =* .749) or No Hint (*r =* .11*, p =* .310) group. The second filler item was also not correlated to the Hint (*r =* .16*, p =* .115) or Not Hint group (*r =* .18*, p =* .093). For block B, hint and no hint groups were moderately correlated *r =* .37*, p* < .001. The first filler item was not correlated to the Hint (*r =* -.04*, p =* .726) but was for the No Hint (*r =* .24*, p =* .017) group. The second filler item was also not correlated to the Hint (*r =* .08*, p =* .456) but was for the No Hint group (*r =* .48*, p <* .001).

**Section 3: Study 2**

Because our power analysis assumed an average inter-item correlation of r = .03, we assessed how close the observed correlations were to that value. Correlations ranged from 0.01 to 0.37 (see table 10). A correlation as low as 0.01 would require an additional 100 participants to calculate the effect as anticipated, while the average correlation among these items (*R* = .22), would only necessitate an additional 30 to 40 people to achieve 90% power. Notably, this sample is still sufficient to achieve at least 80% power.

**Table 10**

*Inter-item Correlations for Time Points*

|  |  | Item 2 | Item 3 | Item 4 | Item 5 |
| --- | --- | --- | --- | --- | --- |
| Item 1 | *R* | 0.041 | -.117* | -.365** | -.315** |
|  | *p* | 0.396 | 0.015 | <.001 | <.001 |
| Item 2 | *R* | 1 | -.103* | -.345** | -.374** |
|  | *p* |  | 0.032 | <.001 | <.001 |
| Item 3 | *R* |  | 1 | -.260** | -.259** |
|  | *p* |  |  | <.001 | <.001 |
| Item 4 | *R* |  |  | 1 | -0.012 |
|  | *p* |  |  |  | 0.81 |

We also compared the within-subjects pairwise analyses of each time point. There was no significant difference among Time 1 (*M* = 3.87, *SE* = 0.11), Time 2 (*M* = 3.91, *SE* = 0.11), or Time 3 (*M* = 3.67, *SE* = 0.11) (all *p*s > .05). Time 4 (*M* = 4.09, *SE* = 0.12) as significantly more positive than Time 3 (*p* = .026) and less than Time 5 (*p* < .001), and Time 5 (*M* = 4.85, *SE* = 0.12) was significantly more positive than all preceding times (all *p*s < .001).

Another way one could assess the degree to which people endorsed more improving or declining narratives is to assess what events they placed where. In Table 10 we organized events based on their positivity absolute valence (the events we determined were positive and negative a priori). We used two criteria for this:

1. Positive narratives identified by positive events in the final two times, stable narratives identified by a mix in of positive and negative in each of the first and last two, and declining narratives identified by positive events in the first two times.
2. Positive narratives identified by both positive events in the final three times, and declining narratives identified by all negative events in the final three times.

**Table 11**

*Placement of Events by Absolute Valence*

|  | Final Two Positive | Perfectly Balanced | Final Two Negative |
| --- | --- | --- | --- |
| Raw Total | 106 | 80 | 46 |
| % of Sample | 26% | 19% | 11% |

Assuming the null hypothesis, that participants are responding randomly, these categorizations should all be equally likely. We conducted a chi-square test to determine whether these values deviated from the null. As expected, there was a significant difference, χ^2^ = 23.41, *p* < .001.

**Table 12**

*Placement of Events by Absolute Valence*

|  | Final Three Positive | Final Three Negative |
| --- | --- | --- |
| Raw Total | 176 | 93 |
| % of Sample | 43% | 23% |

Like the prior analysis, assuming the null hypothesis, that participants are responding randomly, these categorizations should all be equally likely. We conducted a chi-square test to determine whether these values deviated from the null. As expected, and consistent with above method, there was a significant difference, χ^2^ = 25.61, *p* < .001.

This method does, however, conflate our objective ratings with participant’s perceived ratings. An individual may view the neutral event and one of the positive and negative events as equally unvalenced, in which case the placement of that event would be arbitrary to that participant. As such, we have also opted to assess this by grouping the number of participants whose final two events were (on average) more positive than their first two events.

**Table 13**

*Placement of Events by Absolute Valence*

|  | Final Two Positive | Perfectly Balanced | Final Two Negative |
| --- | --- | --- | --- |
| Raw Total | 226 | 26 | 155 |
| % of Sample | 56% | 6% | 38% |

Again, this chi-square test is significant, χ^2^ = 25.61, *p* < .001. Even if we compare improvement against both stability and decline together, improvement is still preferred significantly more, χ^2^ = 4.98, *p* = .026. This analysis gives somewhat different numbers, but the same trend in results, overall, participants tended to place events such that their final two time points were more positive than the initial two time points.

Overall, these results corroborate those in the main manuscript, suggesting when people order events with little to no direction, they generally prefer to do it in such a way that positivity increases over time.

**Section 3: Study 3**

To demonstrate that these perceptions of change may be associated with other beliefs people hold, we also assessed people’s evaluations of Avalon. We used four items each assessing Avalon (e.g., “*I think Avalon is probably a good place to live*” and “*I wouldn’t mind visiting Avalon”*). These items were rated on a 1 (*Strongly Disagree*) to 7 (*Strongly Agree*) scale and demonstrated good reliability (α = .90), so we computed a mean score across the four items.

We then assessed a one-way ANOVA to determine whether the means of each of the conditions differed in their evaluations of Avalon. The omni-bus model was significant *F*(3,390) = 65.31, *p* < .001. Follow up pairwise comparisons were all significant (all *ps* < .001). The pattern of results matched people’s perception of change. The improving town was viewed most favourably (*M* = 6.00, *SD* = 0.83). The town with no additional information was seen as second most desirable (*M* = 5.48, *SD* = 1.06), while the mixed information town was seen less positively (*M* = 4.90, *SD* = 1.09). Lastly, the declining town was seen least positively (*M* = 4.06, *SD* = 1.08).

Overall, this suggests that information about change (or the lack thereof) not only affects people’s perceptions of change, but also their general evaluation of the target. In other words, the improvement default bleeds into other domains, such as people’s general liking of a target. People preferred living in and visiting a town where they knew no information about change than one where they knew mixed information.

**Section 3: Study 4**

In addition to assessing participants’ support for policies, we also assessed to what degree they perceived various equality-enhancing behaviours as important. There was general reliability across these items was acceptable (α = .76).

In line with the analyses conducted in the main paper we assessed these outcomes in a simultaneous regression with past change, future change, and optimism. In this model both beliefs about past change *B* = -0.22, *t*(219) = -4.37, *p* < .001, and future change *B* = 0.20, *t*(219) = 3.64, *p* < .001, but not optimism *B* = 0.03, *t*(219) = 0.57, *p* = .573, predicted support for policies. Again, like with policy support, the unique associations with perceived importance were in opposite, with past improvement predicting less support (consistent with Esposito & Finley, 2009) and future improvement predicting more support (consistent with Peetz et al., 2009).

As with the policies we assessed whether future beliefs may function as a suppressor. Given that these associations demonstrate joint significance (Yzerbyt et al., 2018), we assessed the potential suppression effect of future beliefs on the relationship between past beliefs and policy. A percentile bootstrap confidence interval for the indirect effect (b = 0.11) based on 5000 bootstrapped samples was entirely above zero (0.05 to 0.17) indicating a significant indirect effect.
